# Intestinal permeability in allogeneic hematopoietic stem cell transplantation: A comprehensive review of measurement methods, clinical determinants and prognostic significance

**DOI:** 10.1016/j.htct.2026.106541

**Published:** 2026-09-22

**Authors:** Alexandre Soares Ferreira Junior, Bianca Fernanda Rodrigues da Silva, Nathalia Linares Silva, Mariana Trovao da Silva, Rozana Mesquita Ciconelli, Phillip Scheinberg, Gislane Lelis Vilela de Oliveira

**Affiliations:** a Department of Genetics, Microbiology and Immunology, Institute of Biosciences (IBB), Sao Paulo State University (UNESP), Botucatu, Sao Paulo, Brazil.; b Departamento de Pesquisa da BP-A Beneficência Portuguesa de São Paulo, São Paulo, Brazil.

**Keywords:** Zonulin, Tight junction proteins, Stem cell transplantation, Intestinal permeability, Graft-versus-host disease

## Abstract

The role of intestinal permeability in patients undergoing allogeneic hematopoietic stem cell transplantation remains unclear. This narrative review aimed to critically analyze the available evidence regarding the dynamics, predictors, and prognostic significance of intestinal permeability in the allogeneic hematopoietic stem cell transplantation setting. PubMed was searched from inception to December 2025 to identify relevant studies. Inclusion criteria comprised articles that used any methodology to assess intestinal permeability in the allogeneic hematopoietic stem cell transplantation setting. Animal studies and non-English publications were excluded. Twenty-one studies were identified involving 1,130 patients that used 22 distinct methodologies, encompassing both probe-based and biomarker-based approaches. While most probe-based studies demonstrated significant temporal changes, biomarker-based methodologies yielded conflicting and non-significant findings. Consistent predictors of increased intestinal permeability across the transplantation course included the conditioning regimen, dietary interventions, intestinal microbiota, and immune response. Regarding the prognostic significance, studies reported heterogeneous and often conflicting results across most evaluated clinical outcomes. Although significant advances have been made in understanding the role of intestinal permeability in the allogeneic hematopoietic stem cell transplantation setting, substantial knowledge gaps remain. Future studies should use multiple complementary methodologies simultaneously to capture the different components of the intestinal barrier.

## Introduction

In patients undergoing allogeneic hematopoietic stem cell transplantation (allo-HSCT), intestinal permeability has emerged as a potential prognostic factor to assist in risk stratification and clinical management [1,2]. In the allo-HSCT setting, alterations in intestinal permeability have been investigated as a key underlying mechanism in the complex interplay between host physiology and the intestinal microbiota. This complex interplay has become the focus of extensive research over recent years [3]. Specific patterns of intestinal microbiota disruption, commonly referred to as features of intestinal dysbiosis, have been associated with adverse allo-HSCT-related outcomes [[4], [5], [6]]. For instance, reduced intestinal microbiota diversity has been linked to decreased overall survival, whereas intestinal microbiota domination by a single bacterial taxon has been associated with the development of acute graft-versus-host disease (aGVHD) [4]. In addition, other intestinal microbiota fingerprints have been linked to clinically relevant outcomes, including transplant-related mortality, infectious complications, and *Clostridioides difficile* colitis [4].

Improving allo-HSCT prognosis requires a detailed understanding of the mechanisms underlying the interaction between the intestinal microbiota and the host. Increased intestinal permeability has emerged as a central pathophysiological process modulating this interaction [1,2]. This increase in intestinal permeability may result from injury to the gastrointestinal barrier induced by several factors, including conditioning regimens, antibiotic exposure, and infections. Such injury facilitates the translocation of luminal antigens, dietary components, and microorganisms to the lamina propria and systemic circulation, thereby contributing to the development of allo-HSCT–related outcomes. Although several studies have evaluated the potential prognostic significance of intestinal permeability in the allo-HSCT setting, they are often limited by substantial heterogeneity in the components assessed and in the methodologies used to evaluate intestinal barrier function [[7], [8], [9], [10], [11], [12], [13]]. Moreover, rather than focusing on the dynamics and prognostic implications of intestinal permeability, previous investigations have primarily addressed strategies aimed at preserving barrier integrity [14]. Therefore, there is a clear need for future studies to critically analyze and integrate the current evidence regarding intestinal permeability in this clinical context.

This narrative review aims to provide a comprehensive and critical overview of: 1) the anatomy and physiology of the normal gastrointestinal mucosal barrier; 2) the methodologies used to assess intestinal permeability in the allo-HSCT setting; and 3) the dynamics, modulators, and prognostic significance of intestinal permeability in patients undergoing allo-HSCT.

## Methods

A comprehensive literature search was conducted in PubMed from database inception through December 2025 to identify studies investigating intestinal permeability following allogeneic allo-HSCT. The search strategy was structured around two primary concepts: (1) hematopoietic stem cell transplantation and (2) intestinal permeability (Supplementary Search Strategy). The screening process was conducted by a single reviewer (ASFJ). Inclusion criteria were English-language studies that evaluated any method of assessing intestinal permeability (including both probe-based and biomarker-based approaches) in patients undergoing allo-HSCT. Animal studies were excluded. The search was complemented by manual screening of reference lists from included articles to identify additional relevant studies. Finally, the reference list of a prior review on intestinal permeability in the allo-HSCT setting was also reviewed [14].

Data extraction was conducted by two independent reviewers using template tables. Any disagreements were resolved through discussion between these two reviewers. Among the variables extracted were author and year of publication, intestinal permeability methodologies, dynamics of intestinal permeability throughout allo-HSCT, factors associated with intestinal permeability, and associations between intestinal permeability and clinical outcomes. Although this is not a systematic review, the PRISMA checklist was used as a template, and applicable items were incorporated to improve the transparency of this paper.

## Results and discussion

### Included studies

The search strategy identified a total of 107 records. Following title and abstract screening, 79 studies were excluded. Consequently, 28 studies proceeded to full-text review. An additional 15 studies were excluded after full-text review, most commonly because they did not assess intestinal permeability or were based on animal models. Manual reference searching yielded eight additional articles (Supplementary Table 1) [2,[7], [8], [9], [10], [11],^15^]. Therefore, 21 studies comprising 1,130 patients met the eligibility criteria and were included in this review (Supplementary Table 2). The majority of studies (n = 14; 66.7%) included only adult patients, while six studies (28.6%) included both pediatric and adult populations; none evaluated pediatric patients exclusively. The three main findings of this narrative review are summarized in Figure 1.

**Fig. 1:**
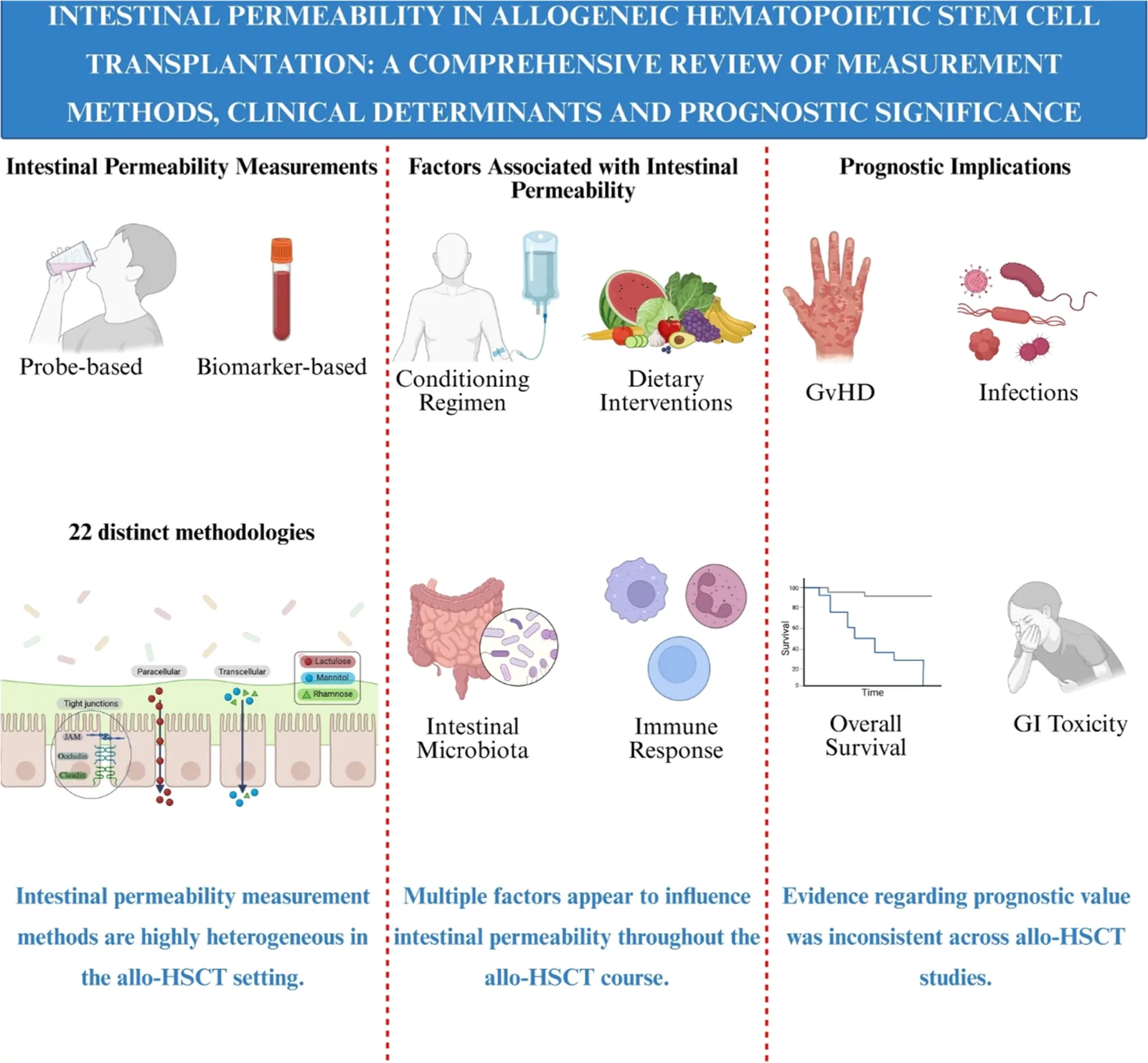
Key findings on intestinal permeability in the allo-HSCT setting. Allo-HSCT: Allogeneic hematopoietic stem cell transplantation;GVHD: Graft versus host disease; GI: Gastrointestinal.

### The healthy gastrointestinal barrier

The gastrointestinal barrier is a complex physical structure that separates the internal environment of the body from the intestinal lumen and exhibits selective permeability, allowing the controlled transport of compounds and microorganisms beneficial to the host while preventing the entry of harmful substances, pathogens, and food antigens [16]. It is primarily composed of three layers: (1) the mucus layer, including inner and outer sublayers that harbor the intestinal microbiota; (2) the intestinal epithelium; and (3) the lamina propria, which contains immune cells [16]. The mucus layer is formed mainly by mucins and commensal microorganisms, including bacteria, fungi, viruses, and parasites [16]. These commensal microorganisms play key roles in maintaining host homeostasis, contributing to digestion, vitamin biosynthesis, metabolite production, and immune system development (helping to distinguish self from non-self antigens) [4,16]. The second layer, the intestinal epithelium, consists of a single layer of specialized cells, including enterocytes, goblet cells, Paneth cells, enteroendocrine cells, and microfold cells [16]. Finally, the third layer, the lamina propria, comprises components of the enteric immune system, including macrophages and dendritic cells.

Microorganisms, metabolites, antigens, and dietary particles present in the intestinal lumen may traverse the gastrointestinal barrier through multiple routes, a process that underlies the concept of intestinal permeability [16,17]. This concept is multifaceted and encompasses alterations in both transcellular pathways, mediated by membrane transporters and endocytic mechanisms, and paracellular routes, which are regulated by tight junctions [16,17]. Tight junctions are protein complexes that regulate paracellular permeability by joining adjacent epithelial cells [16,17]. These tight junction complexes are composed of transmembrane proteins, such as occludin and claudins, and peripheral membrane adaptor proteins, including members of the zonula occludens family, which anchor the junctional complex to the actin cytoskeleton [16,17]. Classically, the paracellular route has been the pathway most closely associated with disease development [17]. However, accumulating evidence in recent years indicates that the transcellular route also plays a critical role in pathogenesis [17]. In the allo-HSCT setting, both transcellular and paracellular routes may be altered, and specific methodological approaches are available to assess each of these pathways [17].

### Intestinal permeability measurement in the allogeneic hematopoietic stem cell transplantation setting

In the allo-HSCT setting, numerous studies have investigated methodologies to quantify intestinal permeability. Overall, approaches to assess intestinal permeability can be broadly categorized into two principal types: 1) Probe-based methods, and 2) Biomarker-based methods. The most commonly used methodologies in the allo-HSCT setting include: 1) Citrulline (eight studies with 342 patients) [[7], [8], [9], [10], [11], [12], [13]]; 2) Lactulose:Rhamnose Ratio (L:R - five studies with 186 patients) [3,10,11,13,15]; 3) Lactulose:Mannitol Ratio (L:M - four studies with 165 patients) [1,12,18,19]; 4) Intestinal fatty acid–binding protein (I-FABP: four studies with 355 patients) [7,8,20,21]; 5) Lipopolysaccharide-binding protein (LBP: four studies with 302 patients) [2,3,13,20]; 6) Chromium-51–labeled ethylenediaminetetraacetic acid absorption (51Cr-EDTA: three studies with 80 patients) [[22], [23], [24]]; 7) Xylose:3-O-methyl-D-glucose (X:O Ratio: three studies with 74 patients) [11,13,15]; 8) Rhamnose:3-O-methyl-D-glucose (R:O Ratio: three studies with 74 patients) [11,13,15]; and 9) Regenerating islet-derived protein 3 alpha (REG3α: two studies with 175 patients). Other methodologies used in one study each included suppression of tumorigenicity (ST2) [3], occludin [3], claudin1 [3], intestinal alkaline phosphatase (IAP) [3], zonulin [1], and calprotectin [1].

Each of these methodologies has specific advantages and limitations (Supplementary Table 3). Overall, probe-based approaches rely on measuring the urinary excretion of orally administered compounds that are poorly absorbed within the gastrointestinal tract [25,26]. These methodologies include: 1) L:M Ratio, 2) L:R Ratio, and 3) the 51-CrEDTA absorption test. Although these are well-established methods that provide a direct functional assessment of intestinal permeability, they typically require active patient participation and may be poorly tolerated in individuals with extensive gastrointestinal tract injury (a common feature in the allo-HSCT setting) [1,3,12,18,27]. Furthermore, when probe-based methodologies are expressed as ratios, false-negative results may occur if absorption of both the paracellular and transcellular probes is increased proportionally, which may give the impression that intestinal permeability is normal [28]. Finally, the permeability route (paracellular vs. transcellular) of many of these probes remains unclear, thereby limiting a comprehensive understanding of the underlying mechanisms of intestinal permeability [17].

In contrast, biomarker-based methodologies represent surrogate measures of intestinal permeability and can be assessed using serum or stool samples. These approaches include citrulline, zonulin, I-FABP, LBP, calprotectin, β-defensin-2, sCD14, myosin light chain kinase 210 isoform (MLCK210), REG3α, ST2, IAP, and claudin-3. A major advantage of biomarker-based methods is their feasibility in clinical practice, as these tests are easy to perform and do not require active patient participation [26,29]. However, further validation and standardization are needed, given the variability in sensitivity and specificity reported across different clinical settings [30,31].

This heterogeneity in intestinal permeability methodologies in the allo-HSCT setting becomes problematic for several reasons, especially considering that none of these studies have properly validated these methodologies against the gold standard methodology (Ussing chambers) [17]. First, these assays assess distinct components of the intestinal barrier and therefore represent different biological concepts [1,8]. For instance, citrulline is a surrogate marker of enterocyte mass, zonulin is a tight junction modulator, REG3α is a surrogate of Paneth cell injury, and I-FABP and I-BABP are surrogates of enterocyte turnover [1,8]. Thus, although they are broadly categorized as intestinal permeability markers, each has distinct regulatory mechanisms and temporal dynamics over the course of allo-HSCT. Second, the gastrointestinal tract is a long and anatomically heterogeneous system, with different barrier characteristics along its length (e.g., small intestine vs. colon). Accordingly, some assays are more specific to certain regions: while I-FABP is expressed in enterocytes of both the small and large intestine, I-BABP is exclusively expressed in the jejunum and ileum [8]. Finally, distinct mechanisms may underlie the increased intestinal permeability at different time points over the course of allo-HSCT. In a study evaluating intestinal permeability in mice undergoing allo-HSCT, different mechanisms were identified throughout the transplant course [32]. Whereas increased intestinal permeability in the early post-transplant period was primarily related to gross epithelial damage, the increase observed during graft-versus-host disease (GvHD) propagation was driven by myosin light chain kinase 210 isoform (MLCK210)-dependent alterations in tight junction permeability [32]. This is further supported by several studies demonstrating conflicting correlations among some of these intestinal permeability surrogates (Supplementary Table 4). Taken together, these findings highlight the need for time-specific selection and validation of intestinal permeability assessment methodologies throughout the allo-HSCT course. Understanding how intestinal permeability evolves over time during allo-HSCT is essential to optimize methodology selection and to interpret results within an appropriate biological and clinical context. In the following section, the current evidence demonstrating intestinal permeability dynamics over the course of allo-HSCT will be further explored.

### Intestinal permeability dynamics over the allo-HSCT

The dynamics of intestinal permeability were evaluated in 18 studies including 834 patients using a total of 21 different methodologies (Table 1). Probe-based methodologies were used in 12 studies involving 425 patients [1,3,[10], [11], [12], [13],^15^,^18^,^19^,[22], [23], [24]]. Furthermore biomarker-based methodologies were used in 13 studies, but evaluated in a total of 657 patients [[1], [2], [3],[7], [8], [9], [10], [11], [12], [13],^20^,^32^]. Across methodologies, studies consistently point toward increased intestinal permeability in the allo-HSCT setting (Supplementary Table 5).

**Table 1:** Dynamics of intestinal permeability after allogeneic hematopoietic stem cell transplantation.

Intestinal Permeability Assay		Author, year n	Intestinal Permeability
Probe-based Methodology	L:R Ratio [3,11,13,15]	Wang 2023 [3] 80	↑ from preconditioning to Day +7, remained elevated at Day +30, and ↓ significantly between Day +7 and Day +30.
		Lutgens 2005 [15] 10	↑ from pre-transplant to post-transplant samples and ↓ between Day +14 and Day +21.
		Blijlevens 2004 [11] 32	↑ from pre-transplant to post-transplant samples and ↓ between Day +14 and Day +21.
		Blijlevens 2005 [13] 32	↑ from pre-transplant to post-transplant samples and ↓ between Day +14 and Day +21.
	L:M Ratio [1,10,12,18,19]	Tyszka 2022 [1] 45	↑ from pre-transplant to Day +7 samples.
		Blijlevens 2005 [10] 32	↑ from preconditioning to Day +7 samples and ↓ between Day +7 and Day +21.
		Borges 2020 [18] 31	↑ from preconditioning to Day +14 and Day +30 samples.
		Da Gama Torres 2008 [19] 53	↑ from admission to Day +6 and Day +14 samples.
		Pontoppidan 2015 [12] 30	↑ from pre-transplant to Day +7 samples. Similar values from pre-transplant to Day 0, Day +14, Day +21 and Day +28 samples.
	X:O Ratio [11,15]	Lutgens 2005 [15] 10	↑ from pre-transplant to Day 0, Day +7, Day +14 and Day +21 samples.
		Blijlevens 2004 [11] 32	↑ from pre-transplant to Day 0, Day +7, Day +14 and Day +21 samples. ↓ from Day +7 to Day +14 and Day +21 samples.
	R:O Ratio [11,15]	Lutgens 2005 [15] 10	↑ from pre-transplant to Day 0, Day +7, Day +14 and Day +21 samples.
		Blijlevens 2004 [11] 32	↑ from pre-transplant to Day +7, Day +14 and Day +21 samples.
	51Cr-EDTA [[22], [23], [24]]	Johansson 1997 [22] 25	↑ from pre-transplant to Day +1, Day +4, Day +7 and Day +10 samples.
		Johansson 2007 [23] 38	In patients with GVHD, intestinal permeability ↑ from baseline to Day −1. Subsequently, patients with grade 2–4 GVHD exhibited a further ↑ from Day −1 to Day +4, whereas permeability remained relatively stable in patients with grade 0–1 GVHD. In both groups, intestinal permeability remained stable at Day +7 and declined by Day +14, approaching baseline levels.
		Johansson 2001 [24] 17	Reduced-intensity conditioning No statistically significant changes in intestinal permeability were observed at any time point relative to baseline in patients receiving RIC. Myeloablative conditioning ↑ from pre-transplant to Day -1, Day +4, Day +7 and Day +14 samples.
Biomarker-based Methodology	REG3α [3,7]	Wang 2023 [3] 80	↑ from preconditioning to. Day +7 samples and ↓ from Day +7 to Day +30.
		Rashidi 2018 [7] 95	↑ at preconditioning samples when compared to healthy controls. ↓ from preconditioning to Day +7, followed by recovery through Day +28.
	ST2 [3]	Wang 2023 [3] 80	↑ from preconditioning to Day +7 and remained stable until Day +30.
	LBP [2,3,20]	Wang 2023 [3] 80	↑ from preconditioning to Day +7. ↓ from Day +7 to Day +30
		Skaarud 2021 [20] 117†	↑ from baseline to Day +21 samples in both the intervention and control groups.
		Blijlevens 2005 [13] 32	↑ from preconditioning to Day 0, Day +7, day +14 and Day +21 samples. Peak at day +7.
		Ye 2012 [2] 73	LBP levels ↑ throughout the allo-HSCT course and remained above baseline through Day +100. In patients with GVHD, levels peaked at Day +14, whereas in patients without GVHD, the peak occurred at Day −1.
	Occludin [3]	Wang 2023 [3] 80	Occludin levels remained similar across preconditioning, Day +7, and Day +30.
	Claudin1 [3]	Wang 2023 [3] 80	↑ from preconditioning to Day +7, then remained stable at Day +30.
	IAP [3]	Wang 2023 [3] 80	↓ from preconditioning to Day +7, with a further ↓ to Day +30.
	I-BABP [8]	Derikx 2009 [8] 29	I-BABP levels decreased significantly over the study period, reaching a nadir at Day +0; however, by Day +21, levels had returned to values comparable to baseline.
	I-FABP [7,8,20]	Skaarud 2021 [20] 117†	↓ from preconditioning to Day +21 samples in both the intervention and control groups.
		Rashidi 2018 [7] 95	↑ at preconditioning samples compared with healthy controls. Patients exhibited an initial ↓ from preconditioning to Day +7, followed by recovery through Day +28.
		Derikx 2009 [8] 29	I-FABP levels decreased significantly over the study period, reaching a nadir at Day +7; however, by Day +21, levels had returned to values comparable to baseline.
	sCD14 [20]	Skaarud 2021 [20] 117†	Levels remained similar from preconditioning to Day +21.
	Fecal Zonulin [1]	Tyszka 2022 [1] 32	↓ from preconditioning to Day +7, Day +14, and Day +21. ↑ at all allo-HSCT time points vs. reference values.
	Beta-defensin-2 [1]	Tyszka 2022 [1] 14	Similar values from preconditioning to Day +7; ↓ at Day +14 and Day +21. ↓ at all allo-HSCT time points vs. reference values.
	Calprotectin [1]	Tyszka 2022 [1] 31	Similar values from preconditioning to Day +7, Day +14 and Day +21. ↓ at all allo-HSCT time points vs. reference values.
	Citrulline [[7], [8], [9], [10], [11], [12], [13],^15^]	Pontoppidan 2015 [12] 30	↓ from preconditioning to Day 0, Day +7, Day +14, day +21, and Day +28.
		Blijlevens 2005 [10] 32	↓ from preconditioning to Day +7 and remained below baseline through Day +21.
		Blijlevens 2004 [11] 32	↓ from preconditioning to Day -6, Day 0, Day +7, Day +14, and Day +21.
		Blijlevens 2005 [13] 32	↓ from preconditioning to Day 0, Day +7, Day +14, and Day +21.
		Lutgens 2005 [15] 10	Overall, citrulline ↓ significantly over time, reaching a nadir at Day +5. Values comparable to baseline by Day +21.
		Rashidi 2018 [7] 95	↓ at preconditioning in allo-HSCT patients vs. healthy controls. ↓ from preconditioning to Day +7, followed by recovery through Day +28.
		Van der Velden 2010 [9] 86	↓ over the allo-HSCT course, remaining below baseline at Day +20. Only patients receiving cyclophosphamide and fludarabine conditioning achieved citrulline levels comparable to baseline by Day +20.
		Derikx 2009 [8] 29	↓ over allo-HSCT, nadir at Day +7, partial recovery by Day +21.
	ZO-1 [32]	Nalle 2019 [32] 9	Patients with GVHD exhibited similar distribution of ZO-1 when compared to healthy controls.
	Actin [32]	Nalle 2019 [32] 9	Patients with GVHD exhibited similar distribution of actin when compared to healthy controls.
	MLCK [32]	Nalle 2019 [32] 9	Patients with GVHD exhibited increased MLCK expression when compared to healthy controls.

51Cr-EDTA Absorption: Chromium-51–labeled ethylenediaminetetraacetic acid absorption; Allo-HSCT: Allogeneic hematopoietic stem cell transplantation; I-FABP: Intestinal fatty acid binding protein; LBP: LPS binding protein; ST2: Suppression of tumorigenicity-2 protein; sCD14: soluble cluster of differentiation; MLCK: long myosin light chain kinase; REG3: Regenerating family member-3 alpha; Not Reported; ZO: Zonula Occludens; †: this study investigated gut barrier function in response to a dietary intervention.

Overall, studies using probe-based methodologies indicate that both transcellular and paracellular permeability increase over the first month following allo-HSCT. Interestingly, patients undergoing allo-HSCT already exhibited increased intestinal permeability prior to the procedure when compared with healthy controls (two studies) [1,18]. This impairment was further exacerbated over time, with a trend toward higher permeability when baseline samples were compared with those obtained at Day 0 (three studies) [11,13,15]. and Day +7 (ten studies) [1,3,[10], [11], [12], [13],^15^,^19^,^22^,^24^]. Most studies also demonstrated a persistent increase in intestinal permeability at approximately Day +14 (seven studies) [11,13,15,18,19,22,24] and Day +30 (two studies) [3,18]. Although permeability began to return toward baseline between Day +21 and Day +30, it generally remained above pre-transplant levels [12].

Biomarker-based methodologies showed heterogeneity in intestinal permeability dynamics, in some cases even yielding conflicting findings. For several indirect biomarkers, such as REG3α, citrulline, ST2, IAP, LBP, I-BABP, and I-FABP, most studies showed trends similar to those observed with probe-based methodologies, with increased permeability between Day +7 and Day +14 and values returning toward baseline around Day +30 [2,3,7,8,[10], [11], [12], [13],^15^,^20^]. In contrast, for other biomarkers, such as occludin and calprotectin, no significant dynamic changes were identified [1,3]. Nevertheless, conflicting patterns were observed for some markers. Zonulin levels were elevated at baseline compared with reference values from a control group; however, relative to baseline, they decreased significantly at Day +7 (76.45 vs. 39.95; p < 0.001), Day +14 (76.45 vs. 43.00; p = NR), and Day +21 (76.45 vs. 41.45; p < 0.001), potentially suggesting improved barrier function [1]. Similarly, β2-defensin levels also decreased over the course of allo-HSCT compared with baseline at Day +7 (17.90 vs. 7.80; p = 0.101), Day +14 (17.90 vs. 4.31; p = NR), and Day +21 (17.90 vs. 6.20; p = 0.006) [1]. These discrepancies highlight the importance of assessing multiple biomarkers and permeability pathways simultaneously in order to obtain a comprehensive perspective [33]. Therefore, future studies should validate and identify the optimal methodologies for evaluating intestinal permeability in the allo-HSCT setting. Such studies should also delineate the main factors driving increased intestinal permeability, as a better understanding of these mechanisms would enable more accurate monitoring of its dynamics throughout the allo-HSCT course.

### Factors associated with increased permeability during the allogeneic hematopoietic stem cell transplantation course

Throughout the allo-HSCT course, several factors may act individually or synergistically to modulate intestinal permeability. Key factors consistently evaluated in allo-HSCT studies include: 1) demographic characteristics (age, sex, underlying diagnosis) [1,3,7]; 2) transplant-related variables (conditioning regimen, use of total body irradiation, time since last chemotherapy) [1,3,7,9,20,21,23,24]; 3) dietary interventions [7,10,13,19,20]; 4) intestinal microbiota [3,20]; and 5) immune response (Table 2) [9,12,13]. Significant associations were found for the following factors: 1) conditioning regimen (four studies) [9,21,23,24], 2) Hematopoietic Cell Transplantation Comorbidity Index score (HCT-CI score: one study) [1], 3) dietary interventions (three studies) [7,10,20], 4) intestinal microbiota (two studies) [3,20] and 5) immune response (three studies) [9,12,13].

**Table 2:** Factors associated with intestinal permeability.

Type	Factor	Author, year n	Findings
Demographics [1,3,7]	Age [1,3,7]	Wang 2023 [3] 80	Baseline L:R ratios were similar regardless of age.
		Tyszka 2022 [1] 45	L:M ratios were similar regardless of age category.
		Rashidi 2018 [7] 95	Age was not associated with baseline levels of citrulline.
			Age was not associated with baseline levels of I-FABP.
			Age was not associated with baseline levels of Reg3α.
	Sex [1,3,7]	Wang 2023 [3] 80	Baseline L:R ratios were similar regardless of sex.
		Tyszka 2022 [1] 45	L:M ratios were similar regardless of sex.
		Rashidi 2018 [7] 95	Sex was not associated with baseline levels of citrulline.
			Sex was not associated with baseline levels of I-FABP.
			Sex was not associated with baseline levels of Reg3α.
	Underlying Diagnosis [3,7]	Wang 2023 [3] 80	Baseline L:R ratios were similar regardless of underlying diagnosis.
		Rashidi 2018 [7] 95	Citrulline levels were not associated with the underlying diagnosis.
			I-FABP levels were not associated with the underlying diagnosis.
			Reg3a levels were not associated with the underlying diagnosis.
Transplant-Related [1,3,7,9,20,21,23,24]	Conditioning Regimen [1,3,9,21,23,24]	Wang 2023 [3] 80	Conditioning regimen did not affect L:R trajectory.
		Tyszka 2022 [1] 45	L:M ratios were similar regardless of conditioning regimen.
		Johansson 2007 [23] 38	51Cr-EDTA was significantly higher in patients receiving myeloablative conditioning than in those receiving RIC on Day +4 and Day +7.
		Van der Velden 2010 [9] 86	Patients receiving myeloablative conditioning showed a rapid decline in citrulline levels within 10 days after initiation of chemotherapy, whereas those receiving non-myeloablative conditioning exhibited an early and short decrease in citrulline levels.
		Johansson 2001 [24] 17	While 51Cr-EDTA did not increase at any time point in patients receiving RIC, a statistically significant increase was consistently observed across all evaluated time points in patients receiving myeloablative conditioning.
		Lounder 2017 [21] 114	I-FABP levels were lower in patients receiving myeloablative regimen.
	Time Since Last Chemotherapy [7]	Rashidi 2018 [7] 95	Time since the last chemotherapy was not correlated with baseline intestinal citrulline levels.
			Time since the last chemotherapy was not correlated with baseline intestinal I-FABP levels.
			Time since the last chemotherapy was not correlated with baseline intestinal Reg3α levels.
	Intensity of Last Anti-Neoplastic Treatment Before Conditioning [7]	Rashidi 2018 [7] 95	Baseline citrulline levels were not associated with the intensity of the last anti-neoplastic treatment before conditioning.
			Baseline I-FABP levels were not associated with the intensity of the last anti-neoplastic treatment before conditioning.
			Baseline Reg3a levels were not associated with the intensity of the last anti-neoplastic treatment before conditioning.
	Total Body Irradiation [3]	Wang 2023 [3] 80	Total body irradiation did not affect L:R trajectory.
	HCT-CI [1,7,20]	Skaarud 2021 [20] 117†	There was no significant association between HCT-CI and baseline I-FABP and Day +21 I-FABP levels.
			There was no significant association between HCT-CI and baseline I-LBP and Day +21 I-LBP levels.
			There was no significant association between HCT-CI and baseline sCD14 and Day +21 sCD14 levels.
		Tyszka 2022 [1] 45	Patients with higher HCT-CI scores demonstrated significantly higher baseline L:M ratios compared with those with lower scores.
		Rashidi 2018 [7] 95	Baseline citrulline levels were not associated with the HCT-CI score.
			Baseline I-FABP levels were not associated with the HCT-CI score.
			Baseline Reg3a levels were not associated with the HCT-CI score.
Dietary [7,10,13,19,20]	Dietary Intervention [7,10,13,19,20]	Skaarud 2021 [20] 117†	Baseline and Day +21 I-FABP levels were not statistically different between patients undergoing a dietary intervention and the control group.
			Baseline and Day +21 LBP levels were not statistically different between patients undergoing a dietary intervention and the control group.
			Day +21 sCD14 levels were higher in patients undergoing a dietary intervention compared with the control group.
		Da Gama Torres 2008 [19] 53	There was no significant difference in intestinal permeability between groups (glutamine vs. control groups).
		Blijlevens 2005 [10] 32	Citrulline levels did not differ significantly between the glutamine and placebo groups at preconditioning and Day +7. However, at Day +21, levels were higher in patients receiving glutamine.
			L:R ratio did not differ significantly between the glutamine and placebo groups.
		Blijlevens 2005 [13] 32	LBP levels did not differ significantly between the glutamine and placebo groups.
		Rashidi 2018 [7] 95	At Day +7, patients receiving TPN between Day 0 and Day +7 had significantly lower citrulline levels compared with those receiving enteral nutrition.
Intestinal Microbiota [3,20]	Intestinal Microbiome Diversity [3,20]	Wang 2023 [3] 80	↑ L:R Ratio in patients with lower Shannon diversity index at Day +7 and Day +30.
		Skaarud 2021 [20] 117†	I-FABP levels were positively correlated with the Shannon index at Day +21 **
			LBP levels were negatively correlated with the Shannon index at Day +21 **
			sCD14 levels were negatively correlated with the Shannon index at Day +21 **
	Microbiota Composition [3,20]	Wang 2023 [3] 80	L:R ratios were significantly associated with the abundance of several bacterial species over the allo-HSCT course.
		Skaarud 2021 [20] 117†	I-FABP levels were positively correlated with *Blautia* abundance at Day +21 **
			LBP levels were not significantly correlated with *Blautia* abundance **
			sCD14 levels were not significantly correlated with *Blautia* abundance.
Immune System [9,12,13]	Inflammatory Markers [9,12,13]	Pontoppidan 2015 [12] 30	Citrulline was negatively associated with IL-6, but not with IL-10, IL-17A, TNF-α, CRP, microRNA-155, or microRNA-146a.
		Van der Velden 2010 [9] 86	Citrulline levels were significantly associated with CRP in multivariable analyses.
		Blijlevens 2005 [13] 32	CRP levels correlated significantly with LBP, the L:R ratio, and citrulline.

51Cr-EDTA Absorption: Chromium-51–labeled ethylenediaminetetraacetic acid absorption; CRP: C Reactive Protein; HCTI-CI: Hematopoietic Cell Transplantation Comorbidity Index; HSCT: Hematopoietic stem cell transplantation; I-FABP: Intestinal fatty acid binding protein; IL: Interleukin; L:M Ratio: Lactulose:Mannitol Ratio; L:R Ratio: Lactulose:Rhamnose Ratio; LBP: LPS binding protein; mL: milliliters; N: Number of patients included in this study; ng: nanograms; NR: Not Reported; NS: Not significant; OTU: Operational Taxonomic Unit; REG3: Regenerating family member-3 alpha; RIC: Reduced intensity conditioning; sCD14: soluble cluster of differentiation; TNF: Tumor necrosis factor; TPN: Total parenteral nutrition; yo: years old; **: only 47 patients provided stool samples for this analysis; †: this study investigated gut barrier function in response to a dietary intervention; ↓: decreased; ↑: increased.

It is important to note that the conflicting or nonsignificant findings related to some of these factors may reflect limitations of the individual studies (such as lack of statistical power due to small sample sizes) as well as limitations of the methodologies themselves. Each of these factors has distinct underlying mechanisms that may affect different components of the gastrointestinal barrier, ultimately resulting in increased intestinal permeability. For instance, conditioning regimens can cause extensive enterocyte injury and increase both transcellular and paracellular permeability [8]. Because both pathways may be simultaneously affected, probe-based methodologies expressed as ratios may fail to detect such changes [8,28]. Indeed, among the six studies that evaluated the impact of conditioning regimens on intestinal permeability, the two that used probe-based ratios did not identify significant associations [1,3]. In contrast, the remaining four studies, which assessed permeability using 51Cr-EDTA, citrulline, and I-FABP, found that myeloablative conditioning was associated with increased intestinal permeability [9,21,23,24]. Taken together, these data emphasize the complexity of assessing intestinal permeability in the allo-HSCT setting. Additionally, as allo-HSCT increasingly shifts toward nonmyeloablative conditioning regimens, there is a clear need to better characterize how intestinal permeability varies according to different chemotherapy protocols. Therefore, future studies should ideally use multiple complementary methodologies to capture the complex and dynamic changes that occur in the gastrointestinal barrier throughout the course of allo-HSCT.

An interesting finding of this review is the consistent association across studies between intestinal microbiota and intestinal permeability [3,20]. In the allo-HSCT setting, the intestinal microbiota has emerged as a key prognostic factor and therapeutic target, with mechanistic studies suggesting that altered intestinal permeability is one of the pathways through which the microbiota influences allo-HSCT outcomes [4,34]. This review identified that both intestinal microbiota diversity indices and microbiota composition are significantly associated with intestinal permeability [3,20]. In an allo-HSCT study including 80 patients, intestinal permeability was assessed using the L:R ratio [3]. An increased L:R ratio was significantly associated with a lower Shannon diversity index at Day +7 (p = 0.044) and Day +30 (p = 0.047). In addition, increased intestinal permeability was associated with a reduction in anaerobic organisms (p = 0.020) and with the presence of several specific bacterial species. Similarly, in another study including 117 patients, the Shannon diversity index was significantly associated with several intestinal permeability biomarkers: 1) I-FABP (baseline: *r* = 0.31; p = 0.043; Day +21: *r* = 0.45; p = 0.005), 2) LBP (Day +21: *r* = −0.35; p = 0.032), and 3) sCD14 (Day +21: *r* = −0.35; p = 0.034) [20]. Furthermore, the abundance of *Blautia* was significantly associated with I-FABP levels on Day +21 (*r* = 0.39; p = 0.017). These data highlight the close interplay between the intestinal microbiota and the gastrointestinal barrier and suggest that future therapeutic strategies targeting intestinal permeability may include modulation of the gut microbiota. Identifying potential therapeutic targets to modulate intestinal permeability is therefore of particular relevance, especially in light of its prognostic significance.

### Prognostic significance of intestinal permeability

The prognostic significance of intestinal permeability in the allo-HSCT setting was evaluated in 14 studies including a total of 868 patients (Table 3) [[1], [2], [3],^7^,^11^,^13^,^15^,^18^,[20], [21], [22], [23],^35^,^36^]. The most frequently assessed allo-HSCT–related outcomes were: 1) GVHD (ten studies with 769 patients) [[1], [2], [3],^7^,^18^,^20^,^21^,^23^,^35^,^36^]; 2) infections (five studies with 387 patients) [[1], [2], [3],^13^,^36^**];** 3) overall survival (four studies with 385 patients) [3,18,20,36]; 4) non-relapse mortality (three studies with 369 patients) [7,20,36]; 5) oral mucositis (three studies with 109 patients) [1,11,13]; and 6) gastrointestinal toxicity (two studies with 35 patients) [15,22]. Additional outcomes included pulmonary complications (one study with 157 patients) [36] and transplant-associated thrombotic microangiopathy (TA-TMA; one study with 80 patients) [3].

**Table 3:** Associations between intestinal permeability and allogeneic hematopoietic stem cell transplantation outcomes.

Outcome	Author, year n	Findings
GvHD [[1], [2], [3],^7^,^18^,^20^,^21^,^23^,^35^,^36^]	Wang 2023 [3] 80	Baseline, Day +7, and Day +30 L:R ratios and zonulin genotypes were not associated with GVHD or gut-GvHD.
	Skaarud 2021 [20] 117†	Baseline and Day +21 I-FABP, LBP and sCD14 were not associated with GVHD.
	Borges 2020 [18] 31	There was no significant association between L:M Ratio and aGVHD.
	Rashidi 2020 [36] 157	There was no significant association between zonulin genotype and the cumulative incidence of aGVHD.
	Tyszka 2022 [1] 45	L:R ratio, zonulin, calprotectin, and beta-defensin-2 measures over the allo-HSCT course were not associated with GVHD, with the exception of calprotectin levels at Day +7.
	Johansson 2007 [23] 38	Patients with mild aGVHD (grade 0–1) exhibited significantly less disruption of intestinal permeability (measured by 51Cr-EDTA) on Day +4 when compared with patients with aGVHD grade 2–4.
	Rashidi 2018 [7] 95	Reg3α, I-FABP and citrulline measures over the allo-HSCT course were not associated with GVHD severity, with the exception of preconditioning citrulline levels.
	Ye 2012 [2] 73	Patients with aGVHD exhibited significantly higher LBP levels at Dy −1, Day +14, Day +21, and Day +28. Additionally, LBP levels were significant associated with the cumulative incidence of aGVHD at Day +7, Day +14, Day +21, and Day +27. An LBP level >15,000 was significantly associated with an increased risk of aGVHD in a multivariable Cox regression model.
	Coron 2021 [35] 19	Ussing chambers assay did not differ significantly between patients with aGVHD and those without aGVHD.
	Lounder 2017 [21 114	I-FABP levels below the median on Day 7 were associated with a higher incidence of GI GVHD.
TA-TMA [3]	Wang 2023 [3] 80	There was no significant association between TA-TMA and the L:R ratio at baseline, Day +7, or Day +30.
Infections [[1], [2], [3],^13^,^36^]	Wang 2023 [3] 80	There was no significant association between BSI and the L:R ratio at baseline, Day +7, or Day +30. Zonulin genotypes were also not associated with BSI.
	Tyszka 2022 [1] 45	There was no significant association between infections and several intestinal permeability markers (L:M ratio, zonulin, calprotectin, and beta-defensin-2) measured at Day −7, Day +7, Day +14, and Day +21, or with the ΔL:M ratio.
	Rashidi 2020 [36] 157	There was no significant association between haptoglobin genotype and the 100-day cumulative incidence of BSI or CDI.
	Blijlevens 2005 [13] 32	There was no significant association between bacteremia and LBP levels at Day -12, and Day 0.
	Ye 2012 [2] 73	In patients with no aGVHD or grade I aGVHD, infections were significantly associated with LBP levels over the allo-HSCT course.
Overall Survival [3,18,20,36]	Wang 2023 [3] 80	Zonulin genotypes were not associated with mortality *
	Borges 2020 [18] 31	There was no significant association between intestinal permeability and mortality.
	Rashidi 2020 [36] 157	There was no significant association between overall survival and haptoglobin genotype.
	Skaarud 2021 [20] 117†	There was no significant association between mortality and several intestinal permeability markers (I-FABP, LBP, and sCD14) measured at baseline and Day +21.
NRM [7,20,36]	Skaarud 2021 [20] 117†	There was no significant association between NRM and several intestinal permeability markers (I-FABP, LBP, and sCD14) measured at baseline and Day +21.
	Rashidi 2020 [36] 157	There was no significant association between NRM and haptoglobin genotype.
	Rashidi 2018 [7] 95	There was a significant association between NRM and Reg3α levels measured at preconditioning, Day +7, and Day +28. No significant association was observed between NRM and I-FABP or citrulline levels.
Oral Mucositis [1,11,13]	Tyszka 2022 [1] 45	There was no significant association between oral mucositis and several intestinal permeability markers (L:M ratio, zonulin, calprotectin, and β-defensin-2) measured at Day −7, Day +7, Day +14, and Day +21. The only exception was zonulin levels at Day +14, which were associated with oral mucositis.
	Blijlevens 2004 [11] 32	There was no significant association between mucositis and any of the probe-based assays (L:R, O:R, and X:R ratios).
	Blijlevens 2005 [13] 32	The peak of oral mucositis coincided with the nadir of citrulline concentrations.
Gastrointestinal toxicity [15,22]	Johansson 1997 [22] 25	There was a significant association between the severity of gastrointestinal toxicity and intestinal permeability. Also, cumulative gastrointestinal toxicity was positively correlated with mean relative intestinal permeability over the 14-day post-HSCT period.
	Lutgens 2005 [15] 10	There was a significant association between gastrointestinal toxicity and citrulline nadir. However, gastrointestinal toxicity was not associated with probe-based methods (L:R, X:O, or R:O ratios).
Pulmonary Complications [36]	Rashidi 2020 [36] 157	There was no significant association between haptoglobin genotype and the 100-day cumulative incidence of pulmonary complications, and cumulative incidence of idiopathic pulmonary syndrome.

Allo-HSCT: Allogeneic hematopoietic stem cell transplantation; BSI: Bloodstream infection; HP: Haptoglobin; I-FABP: Intestinal fatty acid binding protein; LBP: LPS binding protein; sCD14: soluble cluster of differentiation; GVHD: Graft versus host disease; TA-TMA: Transplant associated thrombotic microangiopathy; N: Number of patients; NRM: Non-relapse mortality; *: The zonulin genotype analysis included a different cohort of 116 patients undergoing allo-HSCT; †: this study investigated gut barrier function in response to a dietary intervention.

Although GVHD was the most commonly evaluated outcome, its association with intestinal permeability remains inconsistent. Several studies using diverse permeability assessment methods (e.g., L:R ratio, L:M ratio, zonulin, I-FABP, LBP, sCD14, calprotectin, and Ussing chamber assays) and examining different clinical GVHD endpoints (incidence, gastrointestinal involvement, and severity) reported non-significant findings. Nevertheless, five (50%) of the ten available studies identified potentially relevant associations. For example, in a cohort of 45 patients, intestinal permeability was assessed at Day −7, Day +7, Day +14, and Day +21 using the L:M ratio, fecal zonulin, calprotectin, and β-defensin 2 [1]. In that study, no significant associations were observed between most markers and GVHD across the evaluated time points. However, calprotectin levels at Day +7 were significantly higher in patients who subsequently developed GVHD compared with those who did not (64.758 vs. 14.060; p = 0.040). In another study including 38 patients, those who developed severe GVHD (grade II–IV) exhibited significantly higher intestinal permeability (measured by 51Cr-EDTA) at Day +4 when compared with patients who did not develop this complication (7 vs. 11; p = 0.02) [23]. Preconditioning citrulline levels were also significantly associated with severe GVHD (grade II–IV: p < 0.01; grade III–IV: p = 0.03) in a study including 95 patients [7]. Despite these conflicting findings, the available evidence suggests that intestinal permeability may play a prognostic role in GVHD [1,2,7,21,23]. However, further clarification will require future studies with larger sample sizes and the concomitant assessment of multiple intestinal permeability markers.

Infections represent another clinically relevant endpoint, particularly in light of studies on the intestinal microbiota suggesting that bacterial translocation across the gut barrier may contribute to the development of bloodstream infections (BSI) [4,6,37]. In the allo-HSCT setting, however, among the five studies evaluating this outcome, only one reported a significant association. In that study, which included 73 patients, LBP levels were significantly higher in patients with no or mild GVHD (grade I) who developed infections compared with those who did not (p < 0.001) [2]. In contrast, within the same cohort, LBP levels were not significantly associated with infections among patients with grade II GVHD [2]. Similarly, in the remaining four studies, infections were not significantly associated with other intestinal permeability biomarkers, including the L:R ratio, fecal zonulin, L:M ratio, calprotectin, β-defensin 2, and haptoglobin genotype [1,3,13,36]. For the other clinical outcomes, conflicting and/or predominantly non-significant findings were also reported. With respect to overall survival, all available studies consistently showed no significant association with intestinal permeability [3,18,20,36**].** For non-relapse mortality, among the three included studies, only one identified a significant association between REG3α levels and this outcome at the preconditioning time point (9.91 vs. 6.23; p = 0.02), Day +7 (11.51 vs. 4.20; p < 0.001), and Day +28 (17.33 vs. 10.47; p = 0.03) [7]. Predominantly non-significant associations were also reported for oral mucositis, with only one study demonstrating a significant relationship between fecal zonulin levels and this outcome at Day +14 (41.125 vs. 43.000; p = 0.048)[1]. For pulmonary complications and TA-TMA, no significant associations with intestinal permeability were identified [3,36].

Overall, these findings indicate that although intestinal permeability is frequently altered in patients undergoing allo-HSCT, its prognostic significance remains inconsistent across studies. While several investigations have reported associations between intestinal permeability and allo-HSCT–related outcomes, the available evidence is inconclusive or of limited methodological quality or both. It is possible that, for each specific clinical endpoint, a particular intestinal permeability biomarker may offer greater prognostic accuracy.

### Future directions and conclusions

Although significant advances have been made in understanding the role of intestinal permeability in the allo-HSCT setting, much remains to be elucidated. Clear conclusions regarding the dynamics and prognostic significance of intestinal permeability during allo-HSCT remain elusive due to differences in study design, variability in biomarker assays and sampling time points, lack of adjustment for potential confounders (such as diet, medication use, and comorbidities), and the absence of comparisons with a standardized gold-standard test (Ussing chambers). Notwithstanding these limitations, this comprehensive narrative review highlights the potential role of intestinal permeability as a prognostic variable throughout the allo-HSCT course that can be monitored to guide risk stratification strategies and future therapeutic interventions. To further advance this field, future studies should perform longitudinal assessments using multiple intestinal permeability methodologies to identify the most relevant pathways and modulators of gut barrier function during allo-HSCT. Simultaneous measurement of several permeability biomarkers may better capture the heterogeneity of allo-HSCT patients. In addition, future studies should aim to systematically identify and account for all potential confounders that modulate intestinal permeability in the allo-HSCT setting, both to improve analytical accuracy and to explore them as potential therapeutic targets. Finally, mechanistic studies are needed to further elucidate the pathways through which increased intestinal permeability may influence allo-HSCT-related outcomes, including immune modulation and bacterial translocation.

## Authors contributions

All authors contributed significantly to the development of this study and manuscript. G.L.V.d.O., A.S.F.J., and B.F.R.S. conceived and designed the study with input from other authors. A.S.F.J. performed the literature search, the title and abstract screening and full-text review. A.S.F.J. and B.F.R.S. performed the data abstraction and wrote the first manuscript draft. All authors critically reviewed, edited and collectively approved the final manuscript file.

## Funding Information

This research was funded by the São Paulo Research Foundation (FAPESP), process numbers #2022/12989-6 (Research grant for G.L.V.d.O.); #2023/08142-0 (Fellowship for A.S.F.J.), #2025/07561-5 (Fellowship for B.F.R.S), #2023/12271-0 (Fellowship for N.L.S.); by the Brazilian National Council for Scientific and Technological Development (CNPq), process number #313190/2021-6 (Fellowship for G.L.V.d.O.), and by the Coordenação de Aperfeiçoamento de Pessoal de Nível Superior—Brasil (CAPES)—Finance Code 001. The content in this manuscript is solely the responsibility of the authors and does not in any way represent the official views of the funders.

## Conflicts of interest

The authors declare no conflicts of interest.

## Data Availability

The data that support the findings of this study are available from the corresponding author upon reasonable request.

## Appendix Supplementary materials

Image, application 1
