# Supplementary material for: Intestinal permeability in allogeneic hematopoietic stem cell transplantation: A comprehensive review of measurement methods, clinical determinants and prognostic significance
Source: Hematol Transfus Cell Ther. 2026 Sep 22;48(4):106541. doi: 10.1016/j.htct.2026.106541 (PMC13631814; doi:10.1016/j.htct.2026.106541)
Supplement: Supplementary file 1 [file mmc1.docx]

**Supplementary Files**

**Supplemental Search Strategy**

((((("Intestinal Barrier Function") OR ("Intestinal Permeability")) OR ("Zonuline")) OR ("Gut Barrier")) AND ("Stem Cell Transplantation"[Mesh] OR "Bone Marrow Transplantation"[Mesh] OR (("stem cell"[tiab] OR "stem cells"[tiab] OR "bone marrow"[tiab]) AND (transplant[tiab] OR transplants[tiab] OR transplanted[tiab] OR transplanting[tiab] OR transplantation[tiab] OR transplantations[tiab] OR post-transplant[tiab] OR post-transplants[tiab] OR post-transplantation[tiab] OR post-transplantations[tiab] OR post-transplanted[tiab] OR posttransplant[tiab] OR posttransplants[tiab] OR posttransplanted[tiab] OR posttransplantation[tiab] OR posttransplantations[tiab])) OR ASCT[tiab] OR HSCT[tiab] OR Allo-HSCT[tiab] OR AlloHSCT[tiab]))

**Supplementary Table 1:** Articles identified through manual review of reference lists

| **Author, year** | **Decision** |
| --- | --- |
| Rashidi 2018 [^1^] | Include |
| Ye 2012 [^2^] |  |
| Van der Velden 2010 [^3^] |  |
| Blijlevens 2005 [^4^] |  |
| Lutgens 2005 [^5^] |  |
| Blijlevens 2004 [^6^] |  |
| Derikx 2009 [^7^] |  |
| Blijlevens 2005 [^8^] |  |
| Nalle 2014 [^9^] | Exclude  Animal model study |
| Hill 1998 [^10^] |  |
| Brown 1999 [^11^] |  |
| Noth 2011 [^12^] |  |
| Hanash 2012 [^13^] |  |
| Lehnert 1986 [^14^] |  |
| Lehnert 1994 [^15^] |  |
| Sykes 198 [8 [^16^] |  |
| Koltun 1996 [^17^] |  |
| Takashima 2011 [^18^] |  |
| Lutgens 2003 [^19^] |  |
| Hill 1997 [^20^] |  |
| Keefe 1997 [^21^] | Exclude  Non allo-HSCT study* |
| Sundstrom 1998 [^22^] |  |
| Elting 2003 [^23^] |  |
| Keefe 2000 [^24^] |  |
| Inutsuka 2003 [^25^] |  |
| Fang Yan 2007 [^26^] |  |
| Fang Yan 2011 [^27^] |  |
| Furusawa 2013 [^28^] |  |
| Scott 2020 [^29^] |  |
| Sapone 2006 [^30^] |  |
| Tripathi 2009 [^31^] |  |
| Papp 2008 [^32^] |  |
| Márquez 2012 [^33^] |  |
| Hamarneh 2014 [^34^ ] |  |
| Liu 2016 [^35^] |  |
| Parlato 2018 [^36^] |  |
| Zeissig 2007 [^37^] |  |
| Weber 2008 [^38^] |  |
| Bertiaux-Vandaële 2011 [^39^] |  |
| Marchiando 2011 [^40^] |  |
| Zhao 2016 [^41^] |  |
| Lasek-Bal 2020 [^42^] |  |
| Olsson 2021 [^43^] |  |
| Ellis 2021 [^44^] |  |
| Bjarnason 1983 [^45^] |  |
| Selby 1987 [^46^] |  |
| Jenkins 1992 [^47^] |  |
| Clayburgh 2005 [^48^] |  |
| Rosenblatt 2001 [^49^] |  |
| Madara 1990 [^50^] |  |
| Wang 2005 [^51^] |  |
| Graham 2006 [^52^] |  |
| Al-Sadi 2008 [^53^] |  |
| Garcia 1995 [^54^] |  |
| Garcia 1998 [^55^] |  |
| Smecuol 2001 [^56^] |  |
| Blijlevens 2005 [^57^] |  |
| Meddings 1998 [^58^] |  |
| Blijlevens 2004 [^59^] |  |
| Pytlík 2002 [^60^] |  |
| Lutgens 2004 [^61^] |  |
| Crenn 2008 [^62^] |  |
| Papadopoulou 1996 [^63^ ] |  |
| Ferry 1989 [^64^] |  |
| Taur 2012 [^65^] | Exclude  Intestinal permeability not assessed |
| Montassier 2014 [^66^] |  |
| Slavin 1998 [^67^] |  |
| Khoury 2001 [^68^] |  |
| Storb 1983 [^69^] |  |
| Panoskaltsis-Mortari 1998 [^70^] |  |
| Nestel 1992 [^71^] |  |
| Haak 2018 [^72^] |  |
| Rapoport 1999 [^73^] |  |
| Sonis 2001 [^74^] |  |
| Van Zaanen 1994 [^75^] |  |
| Harris 2016 [^76^] |  |
| Cheney 1987 [^77^] |  |
| Ziegler 1992 [^78^] |  |
| Schloerb 1999 [^79^] |  |
| Tamburini 2018 [^80^] |  |
| Mehta 2017 [^81^] |  |
| Wardill 2012 [^82^] | Exclude  Non-original Research |
| Valitutti 2019 [^83^] |  |
| Lallès 2019 [^84^ ] |  |
| Schulzke 2009 [^85^] |  |
| De Mooij 2017 [^86^] |  |
| Bjarnason 1995 [^87^] |  |
| Bjarnason 1994 [^88^] |  |
| Melichar 2011 [^89^] |  |
| Shallis 2018 [^90^] |  |
| Peled 2016 [^91^] |  |
| Blijlevens 2000 [^92^] |  |
| Hill 2000 [^93^] |  |
| Ferrara 2009 [^94^] |  |
| Tsukita 2001 [^95^] |  |
| Blijlevens 2005 [^96^] |  |
| De-Souza 2005 [^97^] |  |
| Peterson 2004 [^98^] |  |
| Couriel 2004 [^99^] |  |
| Carpenter 2017 [^100^] |  |
| Mahendra 1994 [^101^] | Full text not found |

Allo-HSCT: Allogeneic hematopoietic stem cell transplantation; *: Studies that did not report data specifically for patients undergoing allo-HSCT were also excluded for this reason

**Supplementary Table 2:** General data of studies evaluating intestinal permeability in the allo-HSCT setting

| **Author, year**  **n (Population)** | **Gut Barrier Detection Methods** | **Assessment Timing** |
| --- | --- | --- |
| Wang 2023 [^102^]  80 (Adults and pediatrics) | L:R Ratio, LBP, REG3α, ST2, Occludin, IAP, and Claudin1 | Preconditioning, Day +7, and Day +30 |
| Skaarud 2021 [^103^]  117 (Adults) | I-FABP, LBP, and sCD14 | Baseline, and Day +21 |
| Tyszka 2022 [^104^]  51 (Adults) | L:M Ratio, Zonulin, Calprotectin, and Beta-defensin-2 | L:M Ratio: Day -7, and Day +7  Zonulin, calprotectin and Beta-defensin-2: Day -7, Day +7, Day +14, and Day +21 |
| Pontoppidan 2015 [^105^]  30 (Adults and pediatrics) | L:M Ratio, and Citrulline | Baseline, Day 0, Day +7, Day +14, Day +21, and Day +28 |
| Nalle 2019 [^106^]  9 (NR) | ZO-1, Actin, and MLCK | Two or more weeks after allo-HSCT (specific date NR) |
| Johansson 1997 [^107^]  25 (Adults) | 51Cr-EDTA | Preconditioning, 2D after starting conditioning regimen, Day +1, Day +4, Day +7, Day +10, and Day +14 |
| Johansson 2007 [^108^]  38 (Adults) | 51Cr-EDTA | Preconditioning, Day -1, Day +4, Day +7, and Day +14 |
| Johansson 2001 [^109^]  17 (Adults) | 51Cr-EDTA | Preconditioning, Day -1, Day +4, Day +7, and Day +14 |
| Rashidi 2018 [^1^]  95 (Adults) | Citrulline, I-FABP, and REG3α | Preconditioning, Day +7, and Day +28 |
| Ye 2012 [^2^]  73 (Adults and pediatrics) | LBP | Day -9, Day 0-1, Day +7, Day +14, Day +21, Day +28, Day +56, Day +90-100, At the onset of aGvHD, and at the time of aGvHD remission. |
| Lounder 2017 [^110^]  114 (Adults and pediatrics) | I-FABP | Day +7 |
| Derikx 2009 [^7^]  29 (Adults) | I-FABP, I-BABP, and Citrulline | Day -12, Day -6, Day 0, Day +7, Day +14, and Day +21 |
| Van der Velden 2010 [^3^]  86 (Adults) | Citrulline | Three times weekly from hospital admission to Day +20. |
| Borges 2020 [^111^]  31 (Adults and pediatrics) | L:M Ratio | Preconditioning, Day +14, and Day +30 |
| Coron 2021 [^112^]  19 (Adults) | Ussing chambers assay | Between Day +21 and Day +28 |
| Da Gama Torres 2008 [^113^]  53 (Adults) | L:M Ratio | On admission, Day +6, and Day +14 |
| Rashidi 2020 [^114^]  157 (Adults and pediatrics) | Haptoglobin genotype | Between admission and Day 0 |
| Blijlevens 2005 [^4^]  32 (Adults) | Citrulline, and L:R Ratio | Day -12, Day -6, Day 0, Day +7, Day +14, and Day +21 |
| Lutgens 2005 [^5^]  10 (Adults) | Citrulline*, X:O Ratio, R:O Ratio, and L:R Ratio | Day -12, Day -7, Day 0, Day +7, Day +14, and Day +21 |
| Blijlevens 2004 [^6^]  32 (Adults) | Citrulline, L:R Ratio, R:O Ratio, and X:O Ratio | Preconditioning, Day -12, Day -6, Day 0, Day +7, Day +14, and Day +21 |
| Blijlevens 2005 [^8^]  32 (Adults) | Citrulline, LBP, L:R Ratio, R:O Ratio, and X:O Ratio | Day -12, Day -6, Day 0, Day +7, Day +14, and Day +21 |

51Cr-EDTA Absorption: Chromium-51–labeled ethylenediaminetetraacetic acid absorption; aGvHD: Acute graft-versus-host disease; Allo-HSCT: Allogeneic hematopoietic stem cell transplantation; D: Day; I-BABP: Ileal bile acid–binding protein; I-FABP: Intestinal fatty acid–binding protein; IAP: Intestinal alkaline phosphatase; L: Lactulose; LBP: Lipopolysaccharide-binding protein; M: Mannitol; MLCK210: Myosin light chain kinase 210 isoform; N: Number of patients included in the study; NR: Not reported; O: 3-O-methyl-Day -glucose; R: Rhamnose; REG3α: Regenerating islet-derived protein 3 alpha; sCD14: Soluble cluster of differentiation 14; ST2: Suppression of tumorigenicity 2; X: Xylose; *: citrulline was assessed daily from Day -6 to Day +1, every other day until Day +7, daily until Day +12 and finally on Day +21

**Supplementary Table 3:** Current Methods and Biomarkers for the Assessment of Intestinal Barrier Dysfunction

| **Methodologies** | | **Interpretation** | **Advantages** | **Disadvantages** |
| --- | --- | --- | --- | --- |
| **Probe-based Methodologies** | L:M Ratio [102,104,105,  111,115–117] | ↑ L:M ratio 🡪 ↑ permeability  ↓ L:M ratio 🡪 ↓ permeability | Direct intestinal permeability marker. | Requires the patient to drink an oral solution.  Not appropriate for uncooperative patients (critically ill).  Not well tolerated in patients with nausea or vomiting.  Not suited to evaluate colonic permeability – lactulose and mannitol are degraded by the colonic microbiota.  Controversy regarding the predominant route of mannitol absorption (paracellular vs. transcellular). |
|  | L:R Ratio  [102,115,117,118] | ↑ L:R ratio 🡪 ↑ permeability  ↓ L:R ratio 🡪 ↓ permeability | Direct intestinal permeability marker.  ↓Risk of food-related contamination of results.  ↓LR solution volume and dose.  Well-tolerated and validated in pediatric patients.  Good correlation with enteritis markers. | Not well tolerated in patients with nausea, mucositis or vomiting.  Not suited to evaluate colonic permeability – lactulose is degraded by the colonic microbiota. |
|  | X:O Ratio [5,117,119] | ↑ X:O Ratio 🡪 ↓ permeability  ↓ X:O Ratio 🡪 ↑ permeability | Marker of intestinal absorptive capacity. | Does not evaluate the paracellular route. |
|  | R:O Ratio [5,117,119] | ↑ R:O Ratio 🡪 ↓ permeability  ↓ R:O Ratio 🡪 ↑ permeability | Marker of intestinal absorptive capacity. | This assay does not evaluate the paracellular route. |
|  | 51-CrEDTA Absorption [6,104,108,114,115,  117,120–122] | ↑ Urinary excretion of 51CrEDTA 🡪 ↑ Paracellular pathway impairment. | Marker of total gastrointestinal permeability.  Easily detectable.  Not degraded by colonic bacteria. | Impact on menstrual cycle.  Radioactivity. |
|  | Ussing chambers assay [112,117] | ↓ TEER 🡪 ↑ Intestinal permeability (paracellular route). | Examination of specific intestinal segments separately.  Gold standard technique.  Allows measurement of both paracellular and transcellular route. | May require specialized expertise and equipment.  Time and labor-intensive. |
| **Biomarker-based Methodology** | Citrulline [62,104,105] | ↓ Plasma levels 🡪 ↓ Functional enterocyte mass. | Simple and reliable surrogate of enterocyte mass. | Not valid in cases of renal failure. |
|  | Zonulin [104,115,117,123–125] | ↑ Zonulin 🡪 Impairs enterocyte tight junction function 🡪 ↑ Paracellular transport 🡪 ↑ Permeability. | It is one of the most reliable serum markers for “leaky gut syndrome”. | It is not the sole mediator of paracellular pathway opening.  Concerns regarding the validity and specificity of zonulin ELISA kits.  Gluten, circadian rhythm and the intestinal microbiota can modulate the release of zonulin.  Does not directly assess enterocyte damage.  ↓ Zonulin levels do not necessarily indicate intact barrier integrity. |
|  | I-FABP [7,115,125–128] | ↑ I-FABP 🡪 ↑ Damage to enterocytes of the small and large intestine. | Promising in the early diagnosis of intestinal ischemia. | Can also originate from lesions in other organs.  Does not allow accurate assessment of enterocyte loss in the jejunum and ileum. |
|  | I-BABP [7] | ↑ I-BABP 🡪 ↑ Damage to enterocytes of the jejunum and ileum. | Assessment of jejunum and ileum permeability. | Does not allow for accurate assessment of enterocyte loss in the small and large intestine. |
|  | LBP [115,125,129,130] | ↑ LBP 🡪 ↑ Intestinal permeability. | A reliable indirect marker of intestinal permeability.  Previously explored in other clinical settings. | May be elevated in patients with chronic liver disease and other conditions. |
|  | Calprotectin [104,129,131] | Indirect marker of intestinal permeability.  ↑ Calprotectin 🡪 ↑ intestinal inflammation 🡪 ↑ intestinal permeability. | Sensitive to inflammation of the intestinal mucosa. | Primarily a marker of inflammation rather than intestinal barrier function. |
|  | Beta-defensin-2 [104,132,133] | ↓ Levels 🡪↑ Damage to the intestinal epithelium and disruption of the intestinal barrier. | Marker of intestinal inflammation.  Explored as a predictive marker of GvHD. | Does not directly reflect the integrity of the intestinal barrier. |
|  | sCD14 [125,134,134,135] | ↑ sCD14 🡪 Systemic inflammatory response (immune activation). | Explored as a marker to aid in the early diagnosis and prognostic evaluation of sepsis. | Limitations regarding cutoff values.  Levels may vary in the elderly and patients with renal failure. |
|  | MLCK [106] | Its upregulation leads to increased barrier permeability. | Associated with GVHD pathogenesis. | Requires invasive biopsies. |
|  | REG3α [136,137] | ↑ Plasma levels🡪 ↑ Intestinal injury. | Biomarker of GVHD in the lower GI tract. | Does not directly reflect intestinal permeability. |
|  | ST2 [138,139] | ↑ ST2 🡪 ↑ immune response and ↑ intestinal permeability. | Explored as a predictor of treatment-resistant GvHD and non-relapse mortality. | Reliable cut-off points are still unclear. |
|  | IAP [140] | ↓ IAP 🡪 ↓ Intestinal junction proteins 🡪 ↑ Permeability. | Excellent marker for crypt-villus differentiation. | Influenced by enteral nutritional intake. |
|  | Claudin-3 [141] | Form a dense structure between intestinal epithelial cells to reduce the exchange of permeable material. | A surrogate marker of intestinal permeability. | Indirect measure of intestinal permeability.  Surrogate especially of paracellular route. |
|  | Occludin [102,117] | ↓ Occludin 🡪 ↑ Permeability.  Decreased levels of occludin reflect disorganized tight junctions and loss of epithelial barrier integrity. | Reflects structural damage to the epithelial barrier. | It may not directly reflect functional changes in the barrier. |
|  | Haptoglobin genotype [114,142] | Homozygous HP1-1: not able to produce zonulin.  HP2-1 or HP2-2: able to produce zonulin. | HP2-2 previously associated with chronic GvHD. | Does not allow assessment of the longitudinal dynamics of intestinal permeability. |

51Cr-EDTA Absorption: Chromium-51–labeled ethylenediaminetetraacetic acid absorption; ELISA: Enzyme-linked immunosorbent assay; GI: Gastrointestinal; HP: Haptoglobin; I-BABP: Ileal bile acid–binding protein; I-FABP: Intestinal fatty acid–binding protein; IAP: Intestinal alkaline phosphatase; L:M Ratio: Lactulose:Mannitol ratio; L:R Ratio: Lactulose:Rhamnose ratio; LBP: Lipopolysaccharide-binding protein; LPS: Lipopolysaccharide; MLCK: Myosin light chain kinase 210 isoform; O: 3-O-methyl-Day -glucose; REG3α: Regenerating islet-derived protein 3 alpha; sCD14: Soluble cluster of differentiation 14; ST2: Suppression of tumorigenicity 2; TEER: Transepithelial electrical resistance; X: Xylose; ↑ : Increase; ↓: Decrease

**Supplementary Table 4:** Articles highlighting correlations between intestinal permeability methodologies

| **Author, year**  **n** | **Methodologies** | **Findings** |
| --- | --- | --- |
| Rashidi 2018 [1]  95 | Citruline, I-FABP, and REG3a | There were no correlations between pre-HSCT biomarkers except for a weak correlation between I-FABP and REG3a (r = -0.23; 95% CI 0.03-0.42). |
| Derikx 2009 [7]  29 | I-FABP, and I-BABP | I-FABP and I-BABP levels were significantly correlated (r = 0.436, p < 0.0001). |
| Lutgens 2005 [5]  10 | Citrulline, L:R Ratio, X:O Ratio, and R:O Ratio | Exhibited distinct trajectories.  Citrulline indicated maximal damage at Day +7, whereas probe-based methodologies indicated maximal damage at later time points (Day +14 to Day +21).  Significant correlation only between citrulline and the R:O ratio (p = 0.025). |
| Blijlevens 2004 [6]  32 | Citrulline, L:R Ratio, X:O Ratio, and R:O Ratio | Citrulline levels correlated significantly with the L:R ratio (r = −0.41), X:O ratio (r = 0.24), and R:O ratio (r = 0.41). |
| Wang 2023 [102]  80 | L:R Ratio, LBP, REG3α, ST2, Occludin, IAP, and Claudin1 | The L:R ratio correlated with LBP levels (r² = 0.208; P = 0.0014).  The trajectories of plasma REG3α and ST2 were similar to that of the L:R ratio. |
| Tyszka 2022 [104]  51 | L:M Ratio, Zonulin, Calprotectin, and Beta-defensin-2 | No significant associations were observed between intestinal permeability methodologies. |

HSCT: hematopoietic stem cell transplant; I-BABP: ileal bile acid–binding protein; I-FABP: intestinal fatty acid–binding protein; IAP: intestinal alkaline phosphatase; L:M Ratio: lactulose:mannitol ratio; L:R Ratio: lactulose:rhamnose ratio; LBP: lipopolysaccharide-binding protein; N: number of patients; O: 3-O-methyl-Day -glucose; REG3α: regenerating islet-derived protein 3 alpha; ST2: suppression of tumorigenicity; X: xylose

**Supplementary Table 5:** The Dynamics of Intestinal Permeability over the Allo-HSCT

| **Intestinal Permeability Assay** | | **Author, year**  **n** | **Assessment Timing** | **Intestinal Permeability** |
| --- | --- | --- | --- | --- |
| **Probe-based Methodology** | L:R Ratio [5,6,8,102] | Wang 2023 [102]  80 | Pre-conditioning, Day +7, and Day +30 | Preconditioning vs. Day +7  ↑ L:R ratio on Day +7 (0.19 vs. 0.86; *p* < 0.0001).  Preconditioning vs. Day +30  ↑ L:R ratio on Day +30 (0.19 vs. 0.40; *p* < 0.014).  Day +7 vs. Day +30  ↓ L:R ratio on Day +30 (0.86 vs. 0.40; *p* = 0.014). |
|  |  | Lutgens 2005 [5]  10 | Day -12  Day -7  Day 0  Day +7  Day +14  Day +21 | Day -12: 0.04 (95% CI 0.03-0.06).  Day -7: 0.05 (95% CI 0.03-0.07).  Day 0: 0.13 (95% 0.00-0.27).  Day +7: 0.12 (95% CI 0.06-0.17).  Day +14: 0.18 (95% CI 0.02-0.34).  Day +21: 0.13 (95% CI 0.06-0.20).  All Samples  Compared with baseline values, the ratio was significantly increased at Day +7 (*p* = 0.020) and Day +21 (*p* = 0.012). |
|  |  | Blijlevens 2004 [6]  32 | Pre-conditioning  Day -12  Day -6  Day 0  Day +7  Day +14  Day +21 | Day -12: 0.04 (95% CI 0.04-0.05).  Day -6: 0.04 (95% CI 0.03-0.06).  Day 0: 0.10 (95% CI 0.06-0.14).  Day +7: 0.18 (95% CI 0.14-0.22).  Day +14: 0.20 (95% CI 0.11-0.30).  Day +21: 0.26 (95% CI 0.01-0.51).  Preconditioning vs. Day 0  There was a significant increase in the L:R ratio at Day 0 (*p* < 0.0001). |
|  |  | Blijlevens 2005 [8]  32 | Day -12  Day -6  Day 0  Day +7  Day +14  Day +21 | Day -12: 0.04 (95% CI 0.04-0.05).  Day -6: 0.04 (95% CI 0.03-0.06).  Day 0: 0.10 (95% CI 0.06-0.14).  Day +7: 0.18 (95% CI 0.14-0.22).  Day +14: 0.20 (95% CI 0.11-0.30).  Day +21: 0.26 (95% CI 0.01-0.51).  Preconditioning (Day -12) vs. Day 0  There was a significant increase in the L:R ratio at Day 0 (*p* < 0.0001).  Preconditioning (Day -12) vs. Day +7  There was a significant increase in the L:R ratio at Day +7 (*p* < 0.005).  Preconditioning (Day -12) vs. Day +14  There was a significant increase in the L:R ratio at Day +14 (*p* < 0.005).  Preconditioning (Day -12) vs. Day +21  There was a significant increase in the L:R ratio at Day +21 (*p* < 0.005). |
|  | L:M Ratio [4,104,105, 111,113] | Tyszka 2022 [104]  45 | Day -7  Day +7 | Day -7 vs. Reference values  ↑ L:M ratio when compared to reference values (0.04 vs. 0.035)  ↑ L:M ratio in 53% of patients even before the conditioning regimen (median value 0.190).  Day +7 vs. Reference values  ↑ L:M ratio in 76% of patients on Day +7 (median value 0.242).  Day -7 vs. Day +7  ↑ L:M ratio on Day +7 (0.04 vs. 0.16; *p* <0.01) |
|  |  | Blijlevens 2005 [4]  32 | Pre-conditioning  Day +7  Day +21 | Preconditioning  Glutamine group: 0.04 (±0.02).  Placebo group: 0.04 (±0.02).  Day +7  Glutamine group: 0.15 (±0.07).  Placebo group: 0.19 (±0.08).  Day +21  Glutamine group: 0.11 (±0.14).  Placebo group: 0.17 (±0.13). |
|  |  | Borges 2020 [111]  31 | Pre-conditioning  Day +14  Day +30 | All Samples vs. Controls  Preconditioning: 24% of patients exhibited an L:M ratio exceeding 2 SD above that of healthy control samples.  Day +14: 60% of patients exhibited an L:M ratio exceeding 2 SD above that of healthy control samples.  Day +30: 27% of patients exhibited an L:M ratio exceeding 2 SD above that of healthy control samples.  All Samples  Preconditioning: 0.0127.  Day +14: 0.092.  D:30: 0.141.  Intestinal permeability increased, although the change did not reach statistical significance in a paired Student’s t-test. |
|  |  | Da Gama Torres 2008 [113]  53 | On admission  Day +6  Day +14 | All Samples  Admission: 0.812±0.650  Day +6: 1.460±0.655  Day +14: 1.974±0.878  There were significant rises in the ratio from admission to Day +14 (p < 0.001). |
|  |  | Pontoppidan 2015 [105]  30 | Baseline (7 to 14 days prior to allo-HSCT)  Day 0  Day +7  Day +14  Day +21  Day +28 | Baseline vs. Day 0  Similar L:M Ratios (values NR).  Baseline vs. Day +7  ↑ L:M ratio on Day +7 (values NR, *p* < 0.001)  Baseline vs. Day +14  Similar L:M Ratios (values NR).  Baseline vs. Day +21  Similar L:M Ratios (values NR).  Baseline vs. Day +28  Similar L:M Ratios (values NR). |
|  | X:O Ratio [5,6] | Lutgens 2005 [5]  10 | Day -12  Day -7  Day 0  Day +7  Day +14  Day +21 | Day -12: 0.68 (95% CI 0.56-0.80).  Day -7: 0.65 (95% CI 0.52-0.79).  Day 0: 0.61 (95% CI 0.45-0.77).  Day +7: 0.51 (95% CI 0.47-0.57).  Day +14: 0.51 (95% CI 0.43-0.58).  Day +21: 0.45 (95% CI 0.33-0.58). |
|  |  | Blijlevens 2004 [6]  32 | Pre-conditioning  Day -12  Day -6  Day 0  Day +7  Day +14  Day +21 | Day -12: 0.64 (95% CI 0.59-0.70).  Day -6: 0.59 (95% CI 0.54-0.65).  Day 0: 0.57 (95% CI 0.50-0.63).  Day +7: 0.40 (95% CI 0.31-0.49).  Day +14: 0.44 (95% CI 0.38-0.49).  Day +21: 0.45 (95% CI 0.34-0.57). |
|  | R:O Ratio [5,6] | Lutgens 2005 [5]  10 | Day -12  Day -7  Day 0  Day +7  Day +14  Day +21 | Day -12: 0.23 (95% CI 0.17-0.26).  Day -7: 0.29 (95% CI 0.22-0.35).  Day 0: 0.21 (95% CI 0.15-0.28).  Day +7: 0.13 (95% CI 0.10-0.16).  Day +14: 0.13 (95% CI 0.07-0.20).  Day +21: 0.09 (95% CI 0.07-0.20).  All Samples  Compared with baseline, the R/O ratio was significantly decreased at Day +7 (*p* = 0.027), Day +14 (*p* = 0.033), and Day +21 (*p* = 0.036). |
|  |  | Blijlevens 2004 [6]  32 | Preconditioning  Day -12  Day -6  Day 0  Day +7  Day +14  Day +21 | Day -12: 0.25 (95% CI 0.22-0.27).  Day -6: 0.24 (95% CI 0.21-0.27).  Day 0: 0.25 (95% CI 0.15-0.36).  Day +7: 0.11 (95% CI 0.05-0.18).  Day +14: 0.11 (95% CI 0.07-0.14).  Day +21: 0.11 (95% CI 0.07-0.15). |
|  | 51Cr-EDTA [107–109] | Johansson 1997 [107]  25 | Prior to Conditioning  2d after conditioning regimen initiation  Day +1  Day +4  Day +7  Day +10  Day +14 | Prior to Conditioning vs. 2d after conditioning regiment initiation  ↑ 51Cr-EDTA (p < 0.005).  Prior to Conditioning vs. Day +1  ↑ 51Cr-EDTA (p < 0.005).  Prior to Conditioning vs. Day +4  ↑ 51Cr-EDTA (p < 0.0005).  Prior to Conditioning vs. Day +7  ↑ 51Cr-EDTA (p < 0.0005).  Prior to Conditioning vs. Day +10  ↑ 51Cr-EDTA (p < 0.0005). |
|  |  | Johansson 2007 [108]  38 | Preconditioning, Day -1, Day +4, Day +7, Day +14 | In patients with GvHD, intestinal permeability increased from baseline to D−1. Subsequently, patients with grade 2–4 GvHD exhibited a further increase from D−1 to Day +4, whereas permeability remained relatively stable in patients with grade 0–1 GvHD. In both groups, intestinal permeability remained stable at Day +7 and declined by Day +14, approaching baseline levels. |
|  |  | Johansson 2001 [109]  17 | Preconditioning, Day -1, Day +4, Day +7, Day +14 | Baseline vs. Other Samples  Reduced-intensity conditioning  No statistically significant changes in intestinal permeability were observed at any time point relative to baseline in patients receiving RIC.  Myeloablative conditioning  In patients receiving myeloablative conditioning, a significant increase in intestinal permeability was observed when samples collected on D−1 (1.1 ± 0.3 vs. 2.6 ± 0.7; p < 0.005), Day +4 (1.1 ± 0.3 vs. 3.9 ± 2.6; p < 0.005), Day +7 (1.1 ± 0.3 vs. 4.3 ± 3.2; p < 0.01), and Day +14 (1.1 ± 0.3 vs. 2.6 ± 1.2; p < 0.005) were compared with baseline. |
| **Biomarker-based Methodology** | REG3α [1,102] | Wang 2023 [102]  80 | Preconditioning  Day +7  Day +30 | Preconditioning vs. Day +7  ↑ REG3α (values NR; *p* value <0.001).  Day +7 vs. Day +30  ↓ REG3α (values NR; *p* value <0.01). |
|  |  | Rashidi 2018 [1]  95 | Preconditioning  Day +7  Day +28 | Preconditioning vs. Healthy Controls  Patients exhibited significantly higher baseline Reg3a levels compared with healthy controls (7.07 vs. 4.70; p = 0.006).  All Samples  Patients exhibited an initial decline from preconditioning to Day +7, followed by recovery through Day +28. |
|  | ST2 [102] | Wang 2023 [102]  80 | Preconditioning  Day +7  Day +30 | Preconditioning vs. Day +7  ↑ ST2 (values NR; *p* value <0.001).  Day +7 vs. Day +30  Similar ST2 (values NR; *p* value = 0.58). |
|  | LBP [2,102,103] | Wang 2023 [102]  80 | Preconditioning  Day +7  Day +30 | Preconditioning vs. Day +7  ↑ LBP (values NR; *p* value <0.0001).  Day +7 vs. Day +30  ↓ LBP (values NR; *p* value < 0.0001).  LBP vs. L:R Ratio  There was a significant positive correlation between both markers (r^2^ = 0.208; *p* = 0.0014). |
|  |  | Skaarud 2021 [103]  117† | Baseline (pre-HSCT)  Day +21 | Baseline vs. Day +21  ↑ Intervention group: 13.794 vs. 18.618; *p* < 0.001.  ↑ Control group: 13.389 vs. 18.618; *p* < 0.001. |
|  |  | Blijlevens 2005 [8]  32 | Day -12  Day -6  Day 0  Day +7  Day +14  Day +21 | Day -12: 18.1 (95% CI 14.1-22.1).  Day -6: 16.9 (95% CI 14.0-19.9).  Day 0: 27.1 (95% CI 23.2-31.0).  Day +7: 53.8 (95% CI 43.0-64.7).  Day +14: 36.4 (95% CI 25.1-47.6).  Day +21: 27.0 (95% CI 17.6-36.4).  Preconditioning (Day -12) vs. Day 0  There was a significant increase in LBP levels at Day 0 (p < 0.05).  Preconditioning (Day -12) vs. Day +7  There was a significant increase in LBP levels at Day +7 (p < 0.05).  Preconditioning (Day -12) vs. Day +14  There was a significant increase in LBP levels at Day +14 (p < 0.05).  Preconditioning (Day -12) vs. Day +21  There was a significant increase in LBP levels at Day +21 (p < 0.05). |
|  |  | Ye 2012 [2]  73 | Day -9  Day 0-1  Day +7  Day +14  Day +21  Day +28  Day +56  Day +90-100  At the onset of aGvHD  At the time of complete remission. | All samples  Patients with aGvHD  Day -9: 7989.0  Day -1: 21657.1  Day +7: 18562.7  Day +14: 21966.4  Day +21: 14924.9  Day +28: 16699.7  Day +56: 12128.8  Day +90-Day +100: 13322.5  Overall, LBP levels increased, peaking on Day +14, and remained above baseline levels until Day +100.  Patients without aGvHD  Day -9: 1500.0  Day -1: 12271.2  Day +7: 7057.1  Day +14: 9706.7  Day +21: 8570.2  Day +28: 7140.7  Day +56: 6798.8  Day +90-Day +100: 10609.1  Overall, LBP levels increased, peaking on Day -1, and remained above baseline levels until Day +100. |
|  | Occludin [102] | Wang 2023 [102]  80 | Preconditioning  Day +7  Day +30 | Preconditioning vs. Day +7  Occludin (values NR; *p* value not significant).  Day +7 vs. Day +30  Occludin (values NR; *p* value not significant). |
|  | Claudin1 [102] | Wang 2023 [102]  80 | Preconditioning  Day +7  Day +30 | Preconditioning vs. Day +7  ↑ Claudin1 (values NR; *p* value <0.01).  Day +7 vs. Day +30  Claudin1 (values NR; *p* value < 0.57). |
|  | IAP [102] | Wang 2023 [102]  80 | Preconditioning  Day +7  Day +30 | Preconditioning vs. Day +7  ↓ IAP (values NR; *p* value <0.01).  Day +7 vs. Day +30  ↓ IAP (values NR; *p* value < 0.01). |
|  | I-BABP [7] | Derikx 2009 [7]  29 | Day -12  Day -6  Day 0  Day +7  Day +14  Day +21 | All Samples  I-BABP levels decreased significantly over the study period, reaching a nadir at Day +0; however, by Day +21, levels had returned to values comparable to baseline. |
|  | I-FABP [1,7,103] | Skaarud 2021 [103]  117† | Baseline (pre-HSCT)  Day +21 | Baseline vs. Day +21  ↓ Intervention group: 397 vs. 242pg/ml; *p* = 0.003.  ↓ Control group: 473 vs. 204 pg/ml; *p* < 0.001 |
|  |  | Rashidi 2018 [1]  95 | Preconditioning  Day +7  Day +28 | Preconditioning vs. Healthy Controls  Patients exhibited significantly higher baseline I-FABP levels compared with healthy controls (1.36 vs. 0.93; p = 0.03).  All Samples  Patients exhibited an initial decline from preconditioning to Day +7, followed by recovery through Day +28. |
|  |  | Derikx 2009 [7]  29 | Day -12  Day -6  Day 0  Day +7  Day +14  Day +21 | All Samples  I-FABP levels decreased significantly over the study period, reaching a nadir at Day +7; however, by Day +21, levels had returned to values comparable to baseline. |
|  | sCD14 [103] | Skaarud 2021 [103]  117† | Baseline (pre-HSCT)  Day +21 | Baseline vs. Day +21  Intervention group: 1.651 vs. 21750; *p* = 0.437  Control group: 1820 vs. 1829; *p* = 0.651 |
|  | Fecal Zonulin [104] | Tyszka 2022 [104]  32 | Day -7  Day +7  Day +14  Day +21 | Day -7 vs. Reference values  ↑ Zonulin levels (76.45 vs. <30).  Day +7 vs. Reference values  ↑ Zonulin levels (39.95 vs. <30).  Day +14 vs. Reference values  ↑ Zonulin levels (43.00 vs. <30).  Day +21 vs. Reference values  ↑ Zonulin levels (41.45 vs. <30).  Day -7 vs. Day +7  ↓ Zonulin levels (76.45 vs. 39.95; *p* < 0.001).  Day -7 vs. Day +14  ↓ Zonulin levels (76.45 vs. 43.00; *p* value NR).  Day -7 vs. Day +21  ↓ Zonulin levels (76.45 vs. 41.45; *p* < 0.001).  L:M Ratio vs. Zonulin  There was no significant correlation between L:M Ratio or ∆L:M Ratio and zonulin. |
|  | Beta-defensin-2 [104] | Tyszka 2022 [104]  14 | Day -7  Day +7  Day +14  Day +21 | Day -7 vs. Refence values  ↓ Beta-defensin-2 levels (17.90 vs. <60).  Day +7 vs. Refence values  ↓ Beta-defensin-2 levels (7.80 vs. <60).  Day +14 vs. Refence values  ↓ Beta-defensin-2 levels (4.31 vs. <60).  Day +21 vs. Refence values  ↓ Beta-defensin-2 levels (6.20 vs. <60).  Day -7 vs. Day +7  Similar Beta-defensin-2 levels (17.90 vs. 7.80; *p =* 0.101).  Day -7 vs. Day +14  ↓ Beta-defensin-2 levels (17.90 vs. 4.31; *p* value NR).  Day -7 vs. Day +21  ↓ Beta-defensin-2 levels (17.90 vs. 6.20; *p =* 0.006).  L:M Ratio vs. Beta-defensin-2  There was no significant correlation between L:M Ratio or ∆L:M Ratio and beta-defensin-2. |
|  | Calprotectin [104] | Tyszka 2022 [104]  31 | Day -7  Day +7  Day +14  Day +21 | Day -7 vs. Reference values  ↓ Calprotectin levels (15.50 vs. <50).  Day +7 vs. Reference values  ↓ Calprotectin levels (21.50 vs. <50).  Day +14 vs. Reference values  ↓ Calprotectin levels (19.30 vs. <50).  Day +21 vs. Reference values  ↓ Calprotectin levels (16.90 vs. <50).  Day -7 vs. Day +7  Similar calprotectin levels (15.50 vs. 21.50; *p* = 0.374).  Day -7 vs. Day +14  Similar calprotectin levels (15.50 vs. 19.30; *p* value NR).  Day -7 vs. Day +21  Similar calprotectin levels (15.50 vs. 16.90; *p* = 0.451).  L:M Ratio vs. Calprotectin  There was no significant correlation between L:M Ratio or ∆L:M Ratio and calprotectin. |
|  | Citrulline [1,3–8,105] | Pontoppidan 2015 [105]  30 | Baseline (7 to 14 days prior to allo-HSCT)  Day 0  Day +7  Day +14  Day +21  Day +28 | Baseline vs. Day 0  ↓ Citrulline levels (values NR; *p* < 0.001).  Baseline vs. Day +7  ↓ Citrulline levels (23 ± 1.5 vs. 7.4 ± 1.0 umol/L; *p* < 0.001).  Baseline vs. Day +14  ↓ Citrulline levels (values NR; *p* < 0.001).  Baseline vs. Day +21  ↓ Citrulline levels (values NR; *p* < 0.05).  Baseline vs. Day +28  ↓ Citrulline levels (values NR; *p* < 0.05). |
|  |  | Blijlevens 2005 [4]  32 | Preconditioning  Day +7  Day +21 | Preconditioning  Glutamine group: 34 (±8).  Placebo group: 30 (±7).  Day +7  Glutamine group: 11 (±5).  Placebo group: 10 (±10).  Day +21  Glutamine group: 18 (±6).  Placebo group: 12 (±3). |
|  |  | Blijlevens 2004 [6]  32 | Day -12  Day -6  Day 0  Day +7  Day +14  Day +21 | Day -12: 31.8 (95% CI 28.2-35.3).  Day -6: 25.7 (95% CI 21.7-35.3).  Day 0: 15.1 (95% CI 12.8-17.4).  Day +7: 9.9 (95% CI 7.9-11.8).  Day +14: 14.1 (95% CI 11.2-16.9).  Day +21: 14.5 (95% CI 12.2-16.8).  Preconditioning (Day -12) vs. Day 0  Citrulline levels were significantly decreased at Day 0 compared with baseline (*p* < 0.0001). |
|  |  | Blijlevens 2005 [8]  32 | Day -12  Day -6  Day 0  Day +7  Day +14  Day +21 | Day -12: 31.8 (95% CI 28.2-35.3).  Day -6: 25.7 (95% CI 21.7-35.3).  Day 0: 15.1 (95% CI 12.8-17.4).  Day +7: 9.9 (95% CI 7.9-11.8).  Day +14: 14.1 (95% CI 11.2-16.9).  Day +21: 14.5 (95% CI 12.2-16.8).  Preconditioning (Day -12) vs. Day 0  Citrulline levels were significantly decreased at Day 0 compared with baseline (*p* < 0.0001).  Preconditioning (Day -12) vs. Day +7  Citrulline levels were significantly decreased at Day +7 compared with baseline (*p* < 0.05).  Preconditioning (Day -12) vs. Day +14  Citrulline levels were significantly decreased at Day +14 compared with baseline (*p* < 0.05).  Preconditioning (Day -12) vs. Day +21  Citrulline levels were significantly decreased at Day +21 compared with baseline (*p* < 0.05). |
|  |  | Lutgens 2005 [5]  10 | Daily from Day -6 to Day +1  Every other day until from Day +1 to Day +7  Daily until Day +12  Single sample Day +21 | All Samples  Overall, citrulline concentrations declined significantly over time (p < 0.001), reaching a nadir at Day +5 (mean 8.9; 95% CI 4.4–13.4). Compared with baseline, citrulline levels were significantly lower at all time points, returning to values comparable to baseline by Day +21.  Day -6: 25.7 (95% CI 17.1–34.4).  Day -5: 21.5 (95% CI 14.4–28.6).  Day -4: 18.1 (95% CI 10.3–26.1).  Day -3: 18.6 (95% CI 9.5–27.8).  Day -2: 15.6 (95% CI 9.9–21.2).  Day -1: 14.5 (95% CI 8.4–20.7).  Day 0: 12.3 (95 CI 8.0–16.5).  Day +1: 11.6 (95% CI 6.6-16.6).  Day +3: 9.7 (95% CI 5.3–14.1).  Day +5: 8.9 (95% CI 4.4–13.4).  Day +7: 9.9 (95% CI 5.5–14.2).  Day +8: 11.3 (95% CI 6.3–16.3).  Day +9: 11.5 (95% 6.1–17.0).  Day +10: 12.4 (95% CI 10.5-14.2).  Day +11: 15.7 (95% CI 8.6–22.9).  Day +12: 13.8 (95% CI 6.1–21.4).  Day +21: 20.1 (95% CI 11.1–29.1). |
|  |  | Rashidi 2018 [1]  95 | Preconditioning  Day +7  Day +28 | Preconditioning vs. Healthy Controls  Patients exhibited significantly lower baseline citrulline levels compared with healthy controls (1.88 vs. 10.8; p < 0.01).  All Samples  Patients exhibited an initial decline from preconditioning to Day +7, followed by recovery through Day +28. |
|  |  | Van der Velden 2010 [3]  86 | Three times weekly from hospital admission to Day +20 | All Samples  Regardless of conditioning regimen, patients exhibited a decrease in citrulline levels during allo-HSCT that remained below baseline at Day +20. Only patients receiving cyclophosphamide and fludarabine conditioning achieved citrulline levels comparable to baseline by Day +20. |
|  |  | Derikx 2009 [7]  29 | Day -12  Day -6  Day 0  Day +7  Day +14  Day +21 | All Samples  Citrulline levels declined significantly after allo-HSCT, reaching a nadir on Day +7, followed by partial recovery through Day +21, while remaining below baseline levels. |
|  | ZO-1 [106] | Nalle 2019 [106]  9 | Biopsy was collected at least 2 weeks after allo-HSCT | Patient Sample vs. Controls  Patients with GvHD exhibited similar distribution of ZO-1 when compared to healthy controls subjects. |
|  | Actin [106] | Nalle 2019 [106]  9 | Biopsy was collected at least 2 weeks after allo-HSCT | Patient Sample vs. Controls  Patients with GvHD exhibited similar distribution of actin when compared to healthy controls subjects. |
|  | MLCK [106] | Nalle 2019 [106]  9 | Biopsy was collected at least 2 weeks after allo-HSCT | Patient Sample vs. Controls  Patients with GvHD exhibited increased MLCK expression when compared to healthy controls subject (p < 0.05). |

51Cr-EDTA Absorption: Chromium-51–labeled ethylenediaminetetraacetic acid absorption; Allo-HSCT: Allogeneic hematopoietic stem cell transplantation; D: day; I-FABP: Intestinal fatty acid binding protein; LBP: LPS binding protein; ST2: Suppression of tumorigenicity-2 protein; sCD14: soluble cluster of differentiation; MLCK: long myosin light chain kinase; REG3: Regenerating family member-3 alpha; Not Reported; ZO: Zonula Occludens; †: this study investigated gut barrier function in response to a dietary intervention

**REFERENCES**

1.	Rashidi A, Shanley R, Holtan SG, MacMillan ML, Blazar BR, Khoruts A, et al. Pretransplant Serum Citrulline Predicts Acute Graft-versus-Host Disease. Biology of Blood and Marrow Transplantation. 2018 Nov;24(11):2190–6. doi:10.1016/j.bbmt.2018.06.036

2.	Ye H, Lv M, Zhao X, Zhao X, Huang X. Plasma level of lipopolysaccharide-binding protein is indicative of acute graft-versus-host disease following allogeneic hematopoietic stem cell transplantation. Int J Hematol. 2012 Jun;95(6):680–8. doi:10.1007/s12185-012-1076-z

3.	van der Velden WJFM, Herbers AHE, Feuth T, Schaap NPM, Donnelly JP, Blijlevens NMA. Intestinal damage determines the inflammatory response and early complications in patients receiving conditioning for a stem cell transplantation. PLoS One. 2010 Dec 20;5(12):e15156. doi:10.1371/journal.pone.0015156 PubMed PMID: 21188146; PubMed Central PMCID: PMC3004799.

4.	Blijlevens NMA, Donnelly JP, Naber AHJ, Schattenberg AVMB, dePauw BE. A randomised, double-blinded, placebo-controlled, pilot study of parenteral glutamine for allogeneic stem cell transplant patients. Support Care Cancer. 2005 Oct;13(10):790–6. doi:10.1007/s00520-005-0790-y

5.	Lutgens LCHW, Blijlevens NMA, Deutz NEP, Donnelly JP, Lambin P, De Pauw BE. Monitoring myeloablative therapy‐induced small bowel toxicity by serum citrulline concentration: A comparison with sugar permeability tests. Cancer. 2005 Jan;103(1):191–9. doi:10.1002/cncr.20733

6.	Blijlevens NMA, Lutgens LCHW, Schattenberg AVMB, Donnelly JP. Citrulline: a potentially simple quantitative marker of intestinal epithelial damage following myeloablative therapy. Bone Marrow Transplant. 2004 Aug 1;34(3):193–6. doi:10.1038/sj.bmt.1704563

7.	Derikx JPM, Blijlevens NMA, Donnelly JP, Fujii H, Kanda T, van Bijnen AA, et al. Loss of enterocyte mass is accompanied by diminished turnover of enterocytes after myeloablative therapy in haematopoietic stem-cell transplant recipients. Ann Oncol. 2009 Feb;20(2):337–42. doi:10.1093/annonc/mdn579 PubMed PMID: 18725393.

8.	Blijlevens NMA, Donnelly JP, DePauw BE. Inflammatory response to mucosal barrier injury after myeloablative therapy in allogeneic stem cell transplant recipients. Bone Marrow Transplant. 2005 Oct;36(8):703–7. doi:10.1038/sj.bmt.1705118 PubMed PMID: 16062174.

9.	Nalle SC, Kwak HA, Edelblum KL, Joseph NE, Singh G, Khramtsova GF, et al. Recipient NK cell inactivation and intestinal barrier loss are required for MHC-matched graft-versus-host disease. Sci Transl Med. 2014 Jul 2;6(243):243ra87. doi:10.1126/scitranslmed.3008941 PubMed PMID: 24990882; PubMed Central PMCID: PMC4161673.

10.	Hill GR, Cooke KR, Teshima T, Crawford JM, Keith JC, Brinson YS, et al. Interleukin-11 promotes T cell polarization and prevents acute graft-versus-host disease after allogeneic bone marrow transplantation. J Clin Invest. 1998 Jul 1;102(1):115–23. doi:10.1172/JCI3132 PubMed PMID: 9649564; PubMed Central PMCID: PMC509072.

11.	Brown GR, Lindberg G, Meddings J, Silva M, Beutler B, Thiele D. Tumor necrosis factor inhibitor ameliorates murine intestinal graft-versus-host disease. Gastroenterology. 1999 Mar;116(3):593–601. doi:10.1016/s0016-5085(99)70181-2 PubMed PMID: 10029618.

12.	Noth R, Lange-Grumfeld J, Stüber E, Kruse ML, Ellrichmann M, Häsler R, et al. Increased intestinal permeability and tight junction disruption by altered expression and localization of occludin in a murine graft versus host disease model. BMC Gastroenterol. 2011 Dec;11(1):109. doi:10.1186/1471-230X-11-109

13.	Hanash AM, Dudakov JA, Hua G, O’Connor MH, Young LF, Singer NV, et al. Interleukin-22 protects intestinal stem cells from immune-mediated tissue damage and regulates sensitivity to graft versus host disease. Immunity. 2012 Aug 24;37(2):339–50. doi:10.1016/j.immuni.2012.05.028 PubMed PMID: 22921121; PubMed Central PMCID: PMC3477611.

14.	Lehnert S, Rybka WB, Seemayer TA. Amplification of the graft-versus-host reaction by partial body irradiation. Transplantation. 1986 Jun;41(6):675–9. doi:10.1097/00007890-198606000-00002 PubMed PMID: 3715965.

15.	Lehnert S, Rybka WB. Amplification of the graft-versus-host reaction by cyclophosphamide: dependence on timing of drug administration. Bone Marrow Transplant. 1994 Apr;13(4):473–7. PubMed PMID: 8019473.

16.	Sykes M, Eisenthal A, Sachs DH. Mechanism of protection from graft-vs-host disease in murine mixed allogeneic chimeras. I. Development of a null cell population suppressive of cell-mediated lympholysis responses and derived from the syngeneic bone marrow component. J Immunol. 1988 May 1;140(9):2903–11. PubMed PMID: 2966200.

17.	Koltun WA, Bloomer MM, Colony P, Kauffman GL. Increased intestinal permeability in rats with graft versus host disease. Gut. 1996 Aug;39(2):291–8. doi:10.1136/gut.39.2.291

18.	Takashima S, Kadowaki M, Aoyama K, Koyama M, Oshima T, Tomizuka K, et al. The Wnt agonist R-spondin1 regulates systemic graft-versus-host disease by protecting intestinal stem cells. J Exp Med. 2011 Feb 14;208(2):285–94. doi:10.1084/jem.20101559 PubMed PMID: 21282378; PubMed Central PMCID: PMC3039850.

19.	Lutgens LCHW, Deutz NEP, Gueulette J, Cleutjens JPM, Berger MPF, Wouters BG, et al. Citrulline: A physiologic marker enabling quantitation and monitoring of epithelial radiation-induced small bowel damage. International Journal of Radiation Oncology*Biology*Physics. 2003 Nov;57(4):1067–74. doi:10.1016/S0360-3016(03)00781-8

20.	Hill GR, Crawford JM, Cooke KR, Brinson YS, Pan L, Ferrara JL. Total body irradiation and acute graft-versus-host disease: the role of gastrointestinal damage and inflammatory cytokines. Blood. 1997 Oct 15;90(8):3204–13. PubMed PMID: 9376604.

21.	Keefe DM, Cummins AG, Dale BM, Kotasek D, Robb TA, Sage RE. Effect of high-dose chemotherapy on intestinal permeability in humans. Clin Sci (Lond). 1997 Apr;92(4):385–9. doi:10.1042/cs0920385 PubMed PMID: 9176038.

22.	Sundström GM, Wahlin A, Nordin-Andersson I, Suhr OB. Intestinal permeability in patients with acute myeloid leukemia. Eur J Haematol. 1998 Oct;61(4):250–4. doi:10.1111/j.1600-0609.1998.tb01710.x PubMed PMID: 9820631.

23.	Elting LS, Cooksley C, Chambers M, Cantor SB, Manzullo E, Rubenstein EB. The burdens of cancer therapy: Clinical and economic outcomes of chemotherapy‐induced mucositis. Cancer. 2003 Oct;98(7):1531–9. doi:10.1002/cncr.11671

24.	Keefe DMK. Chemotherapy for cancer causes apoptosis that precedes hypoplasia in crypts of the small intestine in humans. Gut. 2000 Nov 1;47(5):632–7. doi:10.1136/gut.47.5.632

25.	Inutsuka S, Takesue F, Yasuda M, Honda M, Nagahama S, Kusumoto H, et al. Assessment of the Intestinal Permeability following Postoperative Chemotherapy for Human Malignant Disease. Eur Surg Res. 2003;35(1):22–5. doi:10.1159/000067037

26.	Yan F, Cao H, Cover TL, Whitehead R, Washington MK, Polk DB. Soluble proteins produced by probiotic bacteria regulate intestinal epithelial cell survival and growth. Gastroenterology. 2007 Feb;132(2):562–75. doi:10.1053/j.gastro.2006.11.022 PubMed PMID: 17258729; PubMed Central PMCID: PMC3036990.

27.	Yan F, Cao H, Cover TL, Washington MK, Shi Y, Liu L, et al. Colon-specific delivery of a probiotic-derived soluble protein ameliorates intestinal inflammation in mice through an EGFR-dependent mechanism. J Clin Invest. 2011 Jun;121(6):2242–53. doi:10.1172/JCI44031 PubMed PMID: 21606592; PubMed Central PMCID: PMC3104743.

28.	Furusawa Y, Obata Y, Fukuda S, Endo TA, Nakato G, Takahashi D, et al. Commensal microbe-derived butyrate induces the differentiation of colonic regulatory T cells. Nature. 2013 Dec 19;504(7480):446–50. doi:10.1038/nature12721

29.	Scott SA, Fu J, Chang PV. Microbial tryptophan metabolites regulate gut barrier function via the aryl hydrocarbon receptor. Proc Natl Acad Sci USA. 2020 Aug 11;117(32):19376–87. doi:10.1073/pnas.2000047117

30.	Sapone A, de Magistris L, Pietzak M, Clemente MG, Tripathi A, Cucca F, et al. Zonulin upregulation is associated with increased gut permeability in subjects with type 1 diabetes and their relatives. Diabetes. 2006 May;55(5):1443–9. doi:10.2337/db05-1593 PubMed PMID: 16644703.

31.	Tripathi A, Lammers KM, Goldblum S, Shea-Donohue T, Netzel-Arnett S, Buzza MS, et al. Identification of human zonulin, a physiological modulator of tight junctions, as prehaptoglobin-2. Proc Natl Acad Sci U S A. 2009 Sep 29;106(39):16799–804. doi:10.1073/pnas.0906773106 PubMed PMID: 19805376; PubMed Central PMCID: PMC2744629.

32.	Papp M, Foldi I, Nemes E, Udvardy M, Harsfalvi J, Altorjay I, et al. Haptoglobin polymorphism: a novel genetic risk factor for celiac disease development and its clinical manifestations. Clin Chem. 2008 Apr;54(4):697–704. doi:10.1373/clinchem.2007.098780 PubMed PMID: 18258668.

33.	Márquez L, Shen C, Cleynen I, De Hertogh G, Van Steen K, Machiels K, et al. Effects of haptoglobin polymorphisms and deficiency on susceptibility to inflammatory bowel disease and on severity of murine colitis. Gut. 2012 Apr;61(4):528–34. doi:10.1136/gut.2011.240978 PubMed PMID: 21708824.

34.	Hamarneh SR, Mohamed MMR, Economopoulos KP, Morrison SA, Phupitakphol T, Tantillo TJ, et al. A novel approach to maintain gut mucosal integrity using an oral enzyme supplement. Ann Surg. 2014 Oct;260(4):706–14; discussion 714-715. doi:10.1097/SLA.0000000000000916 PubMed PMID: 25203888; PubMed Central PMCID: PMC5034572.

35.	Liu W, Hu D, Huo H, Zhang W, Adiliaghdam F, Morrison S, et al. Intestinal Alkaline Phosphatase Regulates Tight Junction Protein Levels. J Am Coll Surg. 2016 Jun;222(6):1009–17. doi:10.1016/j.jamcollsurg.2015.12.006 PubMed PMID: 27106638; PubMed Central PMCID: PMC5684582.

36.	Parlato M, Charbit-Henrion F, Pan J, Romano C, Duclaux-Loras R, Le Du MH, et al. Human ALPI deficiency causes inflammatory bowel disease and highlights a key mechanism of gut homeostasis. EMBO Mol Med. 2018 Apr;10(4):e8483. doi:10.15252/emmm.201708483 PubMed PMID: 29567797; PubMed Central PMCID: PMC5887907.

37.	Zeissig S, Bürgel N, Günzel D, Richter J, Mankertz J, Wahnschaffe U, et al. Changes in expression and distribution of claudin 2, 5 and 8 lead to discontinuous tight junctions and barrier dysfunction in active Crohn’s disease. Gut. 2007 Jan;56(1):61–72. doi:10.1136/gut.2006.094375 PubMed PMID: 16822808; PubMed Central PMCID: PMC1856677.

38.	Weber CR, Nalle SC, Tretiakova M, Rubin DT, Turner JR. Claudin-1 and claudin-2 expression is elevated in inflammatory bowel disease and may contribute to early neoplastic transformation. Lab Invest. 2008 Oct;88(10):1110–20. doi:10.1038/labinvest.2008.78 PubMed PMID: 18711353; PubMed Central PMCID: PMC2586671.

39.	Bertiaux-Vandaële N, Youmba SB, Belmonte L, Lecleire S, Antonietti M, Gourcerol G, et al. The expression and the cellular distribution of the tight junction proteins are altered in irritable bowel syndrome patients with differences according to the disease subtype. Am J Gastroenterol. 2011 Dec;106(12):2165–73. doi:10.1038/ajg.2011.257 PubMed PMID: 22008894.

40.	Marchiando AM, Shen L, Graham WV, Edelblum KL, Duckworth CA, Guan Y, et al. The epithelial barrier is maintained by in vivo tight junction expansion during pathologic intestinal epithelial shedding. Gastroenterology. 2011 Apr;140(4):1208-1218.e1-2. doi:10.1053/j.gastro.2011.01.004 PubMed PMID: 21237166; PubMed Central PMCID: PMC3066304.

41.	Zhao GJ, Li D, Zhao Q, Lian J, Hu TT, Hong GL, et al. PROGNOSTIC VALUE OF PLASMA TIGHT-JUNCTION PROTEINS FOR SEPSIS IN EMERGENCY DEPARTMENT: AN OBSERVATIONAL STUDY. Shock. 2016 Mar;45(3):326–32. doi:10.1097/SHK.0000000000000524 PubMed PMID: 26863122.

42.	Lasek-Bal A, Kokot A, Gendosz de Carrillo D, Student S, Pawletko K, Krzan A, et al. Plasma Levels of Occludin and Claudin-5 in Acute Stroke Are Correlated with the Type and Location of Stroke but Not with the Neurological State of Patients-Preliminary Data. Brain Sci. 2020 Nov 9;10(11):831. doi:10.3390/brainsci10110831 PubMed PMID: 33182224; PubMed Central PMCID: PMC7695327.

43.	Olsson A, Gustavsen S, Langkilde AR, Hansen TH, Sellebjerg F, Bach Søndergaard H, et al. Circulating levels of tight junction proteins in multiple sclerosis: Association with inflammation and disease activity before and after disease modifying therapy. Mult Scler Relat Disord. 2021 Sep;54:103136. doi:10.1016/j.msard.2021.103136 PubMed PMID: 34247104.

44.	Ellis RJ, Iudicello JE, Heaton RK, Isnard S, Lin J, Routy JP, et al. Markers of Gut Barrier Function and Microbial Translocation Associate with Lower Gut Microbial Diversity in People with HIV. Viruses. 2021 Sep 22;13(10):1891. doi:10.3390/v13101891 PubMed PMID: 34696320; PubMed Central PMCID: PMC8537977.

45.	Bjarnason I, O’Morain C, Levi AJ, Peters TJ. Absorption of 51chromium-labeled ethylenediaminetetraacetate in inflammatory bowel disease. Gastroenterology. 1983 Aug;85(2):318–22. PubMed PMID: 6407889.

46.	Selby PJ, Lopes N, Mundy J, Crofts M, Millar JL, McElwain TJ. Cyclophosphamide priming reduces intestinal damage in man following high dose melphalan chemotherapy. Br J Cancer. 1987 May;55(5):531–3. doi:10.1038/bjc.1987.108 PubMed PMID: 3111515; PubMed Central PMCID: PMC2001721.

47.	Jenkins AP, Nukajam WS, Menzies IS, Creamer B. Simultaneous administration of lactulose and 51Cr-ethylenediaminetetraacetic acid. A test to distinguish colonic from small-intestinal permeability change. Scand J Gastroenterol. 1992 Sep;27(9):769–73. doi:10.3109/00365529209011181 PubMed PMID: 1411284.

48.	Clayburgh DR, Barrett TA, Tang Y, Meddings JB, Van Eldik LJ, Watterson DM, et al. Epithelial myosin light chain kinase-dependent barrier dysfunction mediates T cell activation-induced diarrhea in vivo. J Clin Invest. 2005 Oct;115(10):2702–15. doi:10.1172/JCI24970 PubMed PMID: 16184195; PubMed Central PMCID: PMC1224297.

49.	Rosenblatt J, Raff MC, Cramer LP. An epithelial cell destined for apoptosis signals its neighbors to extrude it by an actin- and myosin-dependent mechanism. Curr Biol. 2001 Nov 27;11(23):1847–57. doi:10.1016/s0960-9822(01)00587-5 PubMed PMID: 11728307.

50.	Madara JL. Maintenance of the macromolecular barrier at cell extrusion sites in intestinal epithelium: physiological rearrangement of tight junctions. J Membr Biol. 1990 Jun;116(2):177–84. doi:10.1007/BF01868675 PubMed PMID: 2380981.

51.	Wang F, Graham WV, Wang Y, Witkowski ED, Schwarz BT, Turner JR. Interferon-gamma and tumor necrosis factor-alpha synergize to induce intestinal epithelial barrier dysfunction by up-regulating myosin light chain kinase expression. Am J Pathol. 2005 Feb;166(2):409–19. doi:10.1016/s0002-9440(10)62264-x PubMed PMID: 15681825; PubMed Central PMCID: PMC1237049.

52.	Graham WV, Wang F, Clayburgh DR, Cheng JX, Yoon B, Wang Y, et al. Tumor necrosis factor-induced long myosin light chain kinase transcription is regulated by differentiation-dependent signaling events. Characterization of the human long myosin light chain kinase promoter. J Biol Chem. 2006 Sep 8;281(36):26205–15. doi:10.1074/jbc.M602164200 PubMed PMID: 16835238.

53.	Al-Sadi R, Ye D, Dokladny K, Ma TY. Mechanism of IL-1beta-induced increase in intestinal epithelial tight junction permeability. J Immunol. 2008 Apr 15;180(8):5653–61. doi:10.4049/jimmunol.180.8.5653 PubMed PMID: 18390750; PubMed Central PMCID: PMC3035485.

54.	Garcia JG, Davis HW, Patterson CE. Regulation of endothelial cell gap formation and barrier dysfunction: role of myosin light chain phosphorylation. J Cell Physiol. 1995 Jun;163(3):510–22. doi:10.1002/jcp.1041630311 PubMed PMID: 7775594.

55.	Garcia JG, Verin AD, Herenyiova M, English D. Adherent neutrophils activate endothelial myosin light chain kinase: role in transendothelial migration. J Appl Physiol (1985). 1998 May;84(5):1817–21. doi:10.1152/jappl.1998.84.5.1817 PubMed PMID: 9572834.

56.	Smecuol E, Bai JC, Sugai E, Vazquez H, Niveloni S, Pedreira S, et al. Acute gastrointestinal permeability responses to different non-steroidal anti-inflammatory drugs. Gut. 2001 Nov;49(5):650–5. doi:10.1136/gut.49.5.650 PubMed PMID: 11600467; PubMed Central PMCID: PMC1728510.

57.	Blijlevens NMA, Donnelly JP, De Pauw BE. Prospective evaluation of gut mucosal barrier injury following various myeloablative regimens for haematopoietic stem cell transplant. Bone Marrow Transplant. 2005 Apr 1;35(7):707–11. doi:10.1038/sj.bmt.1704863

58.	Meddings JB, Gibbons I. Discrimination of site-specific alterations in gastrointestinal permeability in the rat. Gastroenterology. 1998 Jan;114(1):83–92. doi:10.1016/s0016-5085(98)70636-5 PubMed PMID: 9428222.

59.	Blijlevens NMA, Van’T Land B, Donnelly JP, M’Rabet L, De Pauw BE. Measuring mucosal damage induced by cytotoxic therapy. Support Care Cancer. 2004 Apr;12(4):227–33. doi:10.1007/s00520-003-0572-3

60.	Pytlík R, Beneš P, Patorková M, Chocenská E, Gregora E, Procházka B, et al. Standardized parenteral alanyl-glutamine dipeptide supplementation is not beneficial in autologous transplant patients: a randomized, double-blind, placebo controlled study. Bone Marrow Transplant. 2002 Dec 1;30(12):953–61. doi:10.1038/sj.bmt.1703759

61.	Lutgens LCHW, Deutz N, Granzier-Peeters M, Beets-Tan R, De Ruysscher D, Gueulette J, et al. Plasma citrulline concentration: A surrogate end point for radiation-induced mucosal atrophy of the small bowel. A feasibility study in 23 patients. International Journal of Radiation Oncology*Biology*Physics. 2004 Sep;60(1):275–85. doi:10.1016/j.ijrobp.2004.02.052

62.	Crenn P, Messing B, Cynober L. Citrulline as a biomarker of intestinal failure due to enterocyte mass reduction. Clinical Nutrition. 2008 Jun;27(3):328–39. doi:10.1016/j.clnu.2008.02.005

63.	Papadopoulou A, Lloyd DR, Williams MD, Darbyshire PJ, Booth IW. Gastrointestinal and nutritional sequelae of bone marrow transplantation. Arch Dis Child. 1996 Sep;75(3):208–13. doi:10.1136/adc.75.3.208 PubMed PMID: 8976659; PubMed Central PMCID: PMC1511693.

64.	Ferry DM, Butt TJ, Broom MF, Hunter J, Chadwick VS. Bacterial chemotactic oligopeptides and the intestinal mucosal barrier. Gastroenterology. 1989 Jul;97(1):61–7. doi:10.1016/0016-5085(89)91416-9

65.	Taur Y, Xavier JB, Lipuma L, Ubeda C, Goldberg J, Gobourne A, et al. Intestinal Domination and the Risk of Bacteremia in Patients Undergoing Allogeneic Hematopoietic Stem Cell Transplantation. Clinical Infectious Diseases. 2012 Oct 1;55(7):905–14. doi:10.1093/cid/cis580

66.	Montassier E, Batard E, Massart S, Gastinne T, Carton T, Caillon J, et al. 16S rRNA gene pyrosequencing reveals shift in patient faecal microbiota during high-dose chemotherapy as conditioning regimen for bone marrow transplantation. Microb Ecol. 2014 Apr;67(3):690–9. doi:10.1007/s00248-013-0355-4 PubMed PMID: 24402367.

67.	Slavin S, Nagler A, Naparstek E, Kapelushnik Y, Aker M, Cividalli G, et al. Nonmyeloablative stem cell transplantation and cell therapy as an alternative to conventional bone marrow transplantation with lethal cytoreduction for the treatment of malignant and nonmalignant hematologic diseases. Blood. 1998 Feb 1;91(3):756–63. PubMed PMID: 9446633.

68.	Khoury H, Adkins D, Brown R, Pence H, Vij R, Goodnough LT, et al. Low incidence of transplantation-related acute complications in patients with chronic myeloid leukemia undergoing allogeneic stem cell transplantation with a low-dose (550 cGy) total body irradiation conditioning regimen. Biol Blood Marrow Transplant. 2001;7(6):352–8. doi:10.1016/s1083-8791(01)80006-9 PubMed PMID: 11464978.

69.	Storb R, Prentice RL, Buckner CD, Clift RA, Appelbaum F, Deeg J, et al. Graft-versus-host disease and survival in patients with aplastic anemia treated by marrow grafts from HLA-identical siblings. Beneficial effect of a protective environment. N Engl J Med. 1983 Feb 10;308(6):302–7. doi:10.1056/NEJM198302103080602 PubMed PMID: 6337323.

70.	Panoskaltsis-Mortari A, Lacey DL, Vallera DA, Blazar BR. Keratinocyte growth factor administered before conditioning ameliorates graft-versus-host disease after allogeneic bone marrow transplantation in mice. Blood. 1998 Nov 15;92(10):3960–7. PubMed PMID: 9808590.

71.	Nestel FP, Price KS, Seemayer TA, Lapp WS. Macrophage priming and lipopolysaccharide-triggered release of tumor necrosis factor alpha during graft-versus-host disease. J Exp Med. 1992 Feb 1;175(2):405–13. doi:10.1084/jem.175.2.405 PubMed PMID: 1732411; PubMed Central PMCID: PMC2119110.

72.	Haak BW, Littmann ER, Chaubard JL, Pickard AJ, Fontana E, Adhi F, et al. Impact of gut colonization with butyrate producing microbiota on respiratory viral infection following allo-HCT. Blood. 2018 Apr 19;blood-2018-01-828996. doi:10.1182/blood-2018-01-828996

73.	Rapoport AP, Miller Watelet LF, Linder T, Eberly S, Raubertas RF, Lipp J, et al. Analysis of Factors That Correlate With Mucositis in Recipients of Autologous and Allogeneic Stem-Cell Transplants. JCO. 1999 Aug;17(8):2446–2446. doi:10.1200/JCO.1999.17.8.2446

74.	Sonis ST, Oster G, Fuchs H, Bellm L, Bradford WZ, Edelsberg J, et al. Oral Mucositis and the Clinical and Economic Outcomes of Hematopoietic Stem-Cell Transplantation. JCO. 2001 Apr 15;19(8):2201–5. doi:10.1200/JCO.2001.19.8.2201

75.	van Zaanen HC, van der Lelie H, Timmer JG, Fürst P, Sauerwein HP. Parenteral glutamine dipeptide supplementation does not ameliorate chemotherapy-induced toxicity. Cancer. 1994 Nov 15;74(10):2879–84. doi:10.1002/1097-0142(19941115)74:10<2879::aid-cncr2820741022>3.0.co;2-h PubMed PMID: 7954251.

76.	Harris B, Morjaria SM, Littmann ER, Geyer AI, Stover DE, Barker JN, et al. Gut Microbiota Predict Pulmonary Infiltrates after Allogeneic Hematopoietic Cell Transplantation. Am J Respir Crit Care Med. 2016 Aug 15;194(4):450–63. doi:10.1164/rccm.201507-1491OC PubMed PMID: 26886180; PubMed Central PMCID: PMC5003327.

77.	Cheney CL, Abson KG, Aker SN, Lenssen P, Cunningham BA, Buergel NS, et al. Body composition changes in marrow transplant recipients receiving total parenteral nutrition. Cancer. 1987 Apr 15;59(8):1515–9. doi:10.1002/1097-0142(19870415)59:8<1515::aid-cncr2820590821>3.0.co;2-d PubMed PMID: 3102036.

78.	Ziegler TR, Young LS, Benfell K, Scheltinga M, Hortos K, Bye R, et al. Clinical and Metabolic Efficacy of Glutamine-supplemented Parenteral Nutrition after Bone Marrow Transplantation: A Randomized, Double-Blind, Controlled Study. Ann Intern Med. 1992 May 15;116(10):821–8. doi:10.7326/0003-4819-116-10-821

79.	Schloerb PR, Skikne BS. Oral and Parenteral Glutamine in Bone Marrow Transplantation: A Randomized, Double‐Blind Study. J Parenter Enteral Nutr. 1999 May;23(3):117–22. doi:10.1177/0148607199023003117

80.	Tamburini FB, Andermann TM, Tkachenko E, Senchyna F, Banaei N, Bhatt AS. Precision identification of diverse bloodstream pathogens in the gut microbiome. Nat Med. 2018 Dec;24(12):1809–14. doi:10.1038/s41591-018-0202-8

81.	Mehta PA, Davies SM, Leemhuis T, Myers K, Kernan NA, Prockop SE, et al. Radiation-free, alternative-donor HCT for Fanconi anemia patients: results from a prospective multi-institutional study. Blood. 2017 Apr 20;129(16):2308–15. doi:10.1182/blood-2016-09-743112 PubMed PMID: 28179273; PubMed Central PMCID: PMC5766838.

82.	Wardill HR, Bowen JM, Gibson RJ. Chemotherapy-induced gut toxicity: are alterations to intestinal tight junctions pivotal? Cancer Chemother Pharmacol. 2012 Nov;70(5):627–35. doi:10.1007/s00280-012-1989-5 PubMed PMID: 23053271.

83.	Valitutti F, Fasano A. Breaking Down Barriers: How Understanding Celiac Disease Pathogenesis Informed the Development of Novel Treatments. Dig Dis Sci. 2019 Jul;64(7):1748–58. doi:10.1007/s10620-019-05646-y PubMed PMID: 31076989; PubMed Central PMCID: PMC6586517.

84.	Lallès JP. Recent advances in intestinal alkaline phosphatase, inflammation, and nutrition. Nutr Rev. 2019 Oct 1;77(10):710–24. doi:10.1093/nutrit/nuz015 PubMed PMID: 31086953.

85.	Schulzke JD, Ploeger S, Amasheh M, Fromm A, Zeissig S, Troeger H, et al. Epithelial tight junctions in intestinal inflammation. Ann N Y Acad Sci. 2009 May;1165:294–300. doi:10.1111/j.1749-6632.2009.04062.x PubMed PMID: 19538319.

86.	de Mooij CEM, Netea MG, van der Velden WJFM, Blijlevens NMA. Targeting the interleukin-1 pathway in patients with hematological disorders. Blood. 2017 Jun 15;129(24):3155–64. doi:10.1182/blood-2016-12-754994 PubMed PMID: 28483765.

87.	Bjarnason I, MacPherson A, Hollander D. Intestinal permeability: an overview. Gastroenterology. 1995 May;108(5):1566–81. doi:10.1016/0016-5085(95)90708-4 PubMed PMID: 7729650.

88.	Bjarnason I. Intestinal permeability. Gut. 1994 Jan;35(1 Suppl):S18-22. doi:10.1136/gut.35.1_suppl.s18 PubMed PMID: 8125384; PubMed Central PMCID: PMC1378141.

89.	Melichar B, Zezulová M. The significance of altered gastrointestinal permeability in cancer patients. Curr Opin Support Palliat Care. 2011 Mar;5(1):47–54. doi:10.1097/SPC.0b013e328343a043 PubMed PMID: 21326003.

90.	Shallis RM, Terry CM, Lim SH. Changes in intestinal microbiota and their effects on allogeneic stem cell transplantation. American J Hematol. 2018 Jan;93(1):122–8. doi:10.1002/ajh.24896

91.	Peled JU, Hanash AM, Jenq RR. Role of the intestinal mucosa in acute gastrointestinal GVHD. Blood. 2016 Nov 17;128(20):2395–402. doi:10.1182/blood-2016-06-716738

92.	Blijlevens N, Donnelly J, De Pauw B. Mucosal barrier injury: biology, pathology, clinical counterparts and consequences of intensive treatment for haematological malignancy: an overview. Bone Marrow Transplant. 2000 Jun 1;25(12):1269–78. doi:10.1038/sj.bmt.1702447

93.	Hill GR, Ferrara JL. The primacy of the gastrointestinal tract as a target organ of acute graft-versus-host disease: rationale for the use of cytokine shields in allogeneic bone marrow transplantation. Blood. 2000 May 1;95(9):2754–9. PubMed PMID: 10779417.

94.	Ferrara JL, Levine JE, Reddy P, Holler E. Graft-versus-host disease. The Lancet. 2009 May;373(9674):1550–61. doi:10.1016/S0140-6736(09)60237-3

95.	Tsukita S, Furuse M, Itoh M. Multifunctional strands in tight junctions. Nat Rev Mol Cell Biol. 2001 Apr 1;2(4):285–93. doi:10.1038/35067088

96.	Blijlevens NMA. Implications of treatment-induced mucosal barrier injury. Curr Opin Oncol. 2005 Nov;17(6):605–10. PubMed PMID: 16224241.

97.	De-Souza DA, Greene LJ. Intestinal permeability and systemic infections in critically ill patients: Effect of glutamine*: Critical Care Medicine. 2005 May;33(5):1125–35. doi:10.1097/01.CCM.0000162680.52397.97

98.	Peterson DE, Cariello A. Mucosal damage: a major risk factor for severe complications after cytotoxic therapy. Seminars in Oncology. 2004 Jun;31:35–44. doi:10.1053/j.seminoncol.2004.04.006

99.	Couriel D, Caldera H, Champlin R, Komanduri K. Acute graft‐versus‐host disease: Pathophysiology, clinical manifestations, and management. Cancer. 2004 Nov;101(9):1936–46. doi:10.1002/cncr.20613

100.	Carpenter PA. Vitamin A to reduce gut leak and GVHD? Blood. 2017 May 18;129(20):2715–7. doi:10.1182/blood-2017-03-773226

101.	Mahendra P, Bedlow AJ, Ager S, Ancliff PJ, Wraight EP, Marcus RE. Technetium (99mTc)-labelled white cell scanning, 51Cr-EDTA and 14C-mannitol-labelled intestinal permeability studies: non-invasive methods of diagnosing acute intestinal graft-versus-host disease. Bone Marrow Transplant. 1994 Jun;13(6):835–7. PubMed PMID: 7920324.

102.	Wang YM, Abdullah S, Luebbering N, Langenberg L, Duell A, Lake K, et al. Intestinal permeability in patients undergoing stem cell transplantation correlates with systemic acute phase responses and dysbiosis. Blood Advances. 2023 Sep 12;7(17):5137–51. doi:10.1182/bloodadvances.2023009960

103.	Skaarud KJ, Hov JR, Hansen SH, Kummen M, Valeur J, Seljeflot I, et al. Mortality and microbial diversity after allogeneic hematopoietic stem cell transplantation: secondary analysis of a randomized nutritional intervention trial. Sci Rep. 2021 Jun 2;11(1):11593. doi:10.1038/s41598-021-90976-z

104.	Tyszka M, Maciejewska-Markiewicz D, Biliński J, Lubas A, Stachowska E, Basak GW. Increased Intestinal Permeability and Stool Zonulin, Calprotectin and Beta-Defensin-2 Concentrations in Allogenic Hematopoietic Cell Transplantation Recipients. IJMS. 2022 Dec 15;23(24):15962. doi:10.3390/ijms232415962

105.	Pontoppidan PL, Jordan K, Carlsen AL, Uhlving HH, Kielsen K, Christensen M, et al. Associations between gastrointestinal toxicity, micro RNA and cytokine production in patients undergoing myeloablative allogeneic stem cell transplantation. International Immunopharmacology. 2015 Mar;25(1):180–8. doi:10.1016/j.intimp.2014.12.038

106.	Nalle SC, Zuo L, Ong MaLDM, Singh G, Worthylake AM, Choi W, et al. Graft-versus-host disease propagation depends on increased intestinal epithelial tight junction permeability. Journal of Clinical Investigation. 2019 Jan 22;129(2):902–14. doi:10.1172/JCI98554

107.	Johansson JE, Ekman T. Gastro-intestinal toxicity related to bone marrow transplantation: disruption of the intestinal barrier precedes clinical findings. Bone Marrow Transplant. 1997 May 1;19(9):921–5. doi:10.1038/sj.bmt.1700765

108.	Johansson JE, Ekman T. Gut Toxicity During Hemopoietic Stem Cell Transplantation May Predict Acute Graft-Versus-Host Disease Severity in Patients. Dig Dis Sci. 2007 Aug 7;52(9):2340–5. doi:10.1007/s10620-006-9404-x

109.	Johansson JE, Brune M, Ekman T. The gut mucosa barrier is preserved during allogeneic, haemopoietic stem cell transplantation with reduced intensity conditioning. Bone Marrow Transplant. 2001 Oct 1;28(8):737–42. doi:10.1038/sj.bmt.1703230

110.	Lounder DT, Khandelwal P, Dandoy CE, Jodele S, Grimley MS, Wallace G, et al. Lower levels of vitamin A are associated with increased gastrointestinal graft-versus-host disease in children. Blood. 2017 May 18;129(20):2801–7. doi:10.1182/blood-2017-02-765826

111.	Borges LR, Vilela EE, Ferrari MDLA, Cunha AS, Vasconcelos AC, Torres HOG. Diagnosis of acute graft-versus-host disease in the gastrointestinal tract of patients undergoing allogeneic hematopoietic stem cell transplantation. A descriptive and critical study of diagnostic tests. Hematology, Transfusion and Cell Therapy. 2020 Jul;42(3):245–51. doi:10.1016/j.htct.2019.07.002

112.	Coron E, Esnaud E, Chevallier P, Bessard A, Perez Cuadrado‐Robles E, David G, et al. Early remodeling of the colonic mucosa after allogeneic hematopoietic stem cells transplantation: An open‐label controlled pilot study on 19 patients. UEG Journal. 2021 Oct;9(8):955–63. doi:10.1002/ueg2.12128

113.	Da Gama Torres HO, Vilela EG, Da Cunha AS, Goulart EMA, Souza MHC, Aguirre ACC, et al. Efficacy of glutamine-supplemented parenteral nutrition on short-term survival following allo-SCT: a randomized study. Bone Marrow Transplant. 2008 Jun;41(12):1021–7. doi:10.1038/bmt.2008.27

114.	Rashidi A, Allen T, Shanley R, Ebadi M, Weisdorf DJ. Haptoglobin genotype and gut barrier-related complications of allogeneic hematopoietic cell transplantation. Bone Marrow Transplant. 2020 Feb;55(2):464–6. doi:10.1038/s41409-019-0540-2

115.	Vanuytsel T, Tack J, Farre R. The Role of Intestinal Permeability in Gastrointestinal Disorders and Current Methods of Evaluation. Front Nutr. 2021 Aug 26;8:717925. doi:10.3389/fnut.2021.717925

116.	Odenwald MA, Turner JR. Intestinal permeability defects: is it time to treat? Clin Gastroenterol Hepatol. 2013 Sep;11(9):1075–83. doi:10.1016/j.cgh.2013.07.001 PubMed PMID: 23851019; PubMed Central PMCID: PMC3758766.

117.	Mønsted MØ, Falck ND, Pedersen K, Buschard K, Holm LJ, Haupt-Jorgensen M. Intestinal permeability in type 1 diabetes: An updated comprehensive overview. Journal of Autoimmunity. 2021 Aug;122:102674. doi:10.1016/j.jaut.2021.102674

118.	Malagon I, Onkenhout W, Klok M, van der Poel PFH, Bovill JG, Hazekamp MG. Rhamnose and rhamnitol in dual sugar permeability tests. J Pediatr Gastroenterol Nutr. 2006 Aug;43(2):265–6. doi:10.1097/01.mpg.0000226379.41365.62 PubMed PMID: 16877999.

119.	Zuckerman MJ, Menzies IS, Ho H, Gregory GG, Casner NA, Crane RS, et al. Assessment of Intestinal Permeability and Absorption in Cirrhotic Patients with Ascites Using Combined Sugar Probes. Dig Dis Sci. 2004 Apr;49(4):621–6. doi:10.1023/B:DDAS.0000026307.56909.21

120.	Maccioni L, Gao B, Leclercq S, Pirlot B, Horsmans Y, De Timary P, et al. Intestinal permeability, microbial translocation, changes in duodenal and fecal microbiota, and their associations with alcoholic liver disease progression in humans. Gut Microbes. 2020 Nov 9;12(1):1782157. doi:10.1080/19490976.2020.1782157 PubMed PMID: 32588725; PubMed Central PMCID: PMC7524402.

121.	Grootjans J, Thuijls G, Verdam F, Derikx JP, Lenaerts K, Buurman WA. Non-invasive assessment of barrier integrity and function of the human gut. World J Gastrointest Surg. 2010 Mar 27;2(3):61–9. doi:10.4240/wjgs.v2.i3.61 PubMed PMID: 21160852; PubMed Central PMCID: PMC2999221.

122.	Acharya B, Tofthagen M, Maciej-Hulme ML, Suissa MR, Karlsson NG. Limited support for a direct connection between prebiotics and intestinal permeability - a systematic review. Glycoconj J. 2024 Oct;41(4–5):323–42. doi:10.1007/s10719-024-10165-8 PubMed PMID: 39287885; PubMed Central PMCID: PMC11522178.

123.	Malíčková K, Francová I, Lukáš M, Kolář M, Králíková E, Bortlík M, et al. Fecal zonulin is elevated in Crohn’s disease and in cigarette smokers. Pract Lab Med. 2017 Dec;9:39–44. doi:10.1016/j.plabm.2017.09.001 PubMed PMID: 29034305; PubMed Central PMCID: PMC5633835.

124.	Hałasa M, Maciejewska D, Ryterska K, Baśkiewicz-Hałasa M, Safranow K, Stachowska E. Assessing the Association of Elevated Zonulin Concentration in Stool with Increased Intestinal Permeability in Active Professional Athletes. Medicina (Kaunas). 2019 Oct 21;55(10):710. doi:10.3390/medicina55100710 PubMed PMID: 31640273; PubMed Central PMCID: PMC6843915.

125.	Bibolar AC, Crecan-Suciu BD, Păunescu RL, Nechita VI, Verisezan-Roșu O, Micluția IV. Intestinal Permeability and Depression—A Narrative Review of Selected Blood-Based Biomarkers. IJMS. 2025 Oct 16;26(20):10076. doi:10.3390/ijms262010076

126.	Matsumoto S, Sekine K, Funaoka H, Yamazaki M, Shimizu M, Hayashida K, et al. Diagnostic performance of plasma biomarkers in patients with acute intestinal ischaemia. Br J Surg. 2014 Feb;101(3):232–8. doi:10.1002/bjs.9331 PubMed PMID: 24402763.

127.	Sarikaya M, Ergül B, Doğan Z, Filik L, Can M, Arslan L. Intestinal fatty acid binding protein (I-FABP) as a promising test for Crohn’s disease: a preliminary study. Clin Lab. 2015;61(1–2):87–91. doi:10.7754/clin.lab.2014.140518 PubMed PMID: 25807642.

128.	Kitai T, Kim YH, Kiefer K, Morales R, Borowski AG, Grodin JL, et al. Circulating intestinal fatty acid-binding protein (I-FABP) levels in acute decompensated heart failure. Clin Biochem. 2017 Jun;50(9):491–5. doi:10.1016/j.clinbiochem.2017.02.014 PubMed PMID: 28232029; PubMed Central PMCID: PMC5474350.

129.	Seethaler B, Basrai M, Neyrinck AM, Nazare JA, Walter J, Delzenne NM, et al. Biomarkers for assessment of intestinal permeability in clinical practice. Am J Physiol Gastrointest Liver Physiol. 2021 Jul 1;321(1):G11–7. doi:10.1152/ajpgi.00113.2021 PubMed PMID: 34009040.

130.	Utrata A, Schmidtner N, Mester P, Schmid S, Müller M, Pavel V, et al. Plasma Lipopolysaccharide-Binding Protein (LBP) Is Induced in Critically Ill Females with Gram-Negative Infections-Preliminary Study. Infect Dis Rep. 2025 Jan 28;17(1):10. doi:10.3390/idr17010010 PubMed PMID: 39997462; PubMed Central PMCID: PMC11855555.

131.	Jukic A, Bakiri L, Wagner EF, Tilg H, Adolph TE. Calprotectin: from biomarker to biological function. Gut. 2021 Oct;70(10):1978–88. doi:10.1136/gutjnl-2021-324855 PubMed PMID: 34145045; PubMed Central PMCID: PMC8458070.

132.	Fusco A, Savio V, Donniacuo M, Perfetto B, Donnarumma G. Antimicrobial Peptides Human Beta-Defensin-2 and -3 Protect the Gut During Candida albicans Infections Enhancing the Intestinal Barrier Integrity: In Vitro Study. Front Cell Infect Microbiol. 2021;11:666900. doi:10.3389/fcimb.2021.666900 PubMed PMID: 34178720; PubMed Central PMCID: PMC8223513.

133.	August KJ, Chiang KY, Qayed M, Dulson A, Worthington-White D, Cole CR, et al. Relative defects in mucosal immunity predict acute graft-versus-host disease. Biol Blood Marrow Transplant. 2014 Jul;20(7):1056–9. doi:10.1016/j.bbmt.2014.03.012 PubMed PMID: 24641826.

134.	Chenevier-Gobeaux C, Borderie D, Weiss N, Mallet-Coste T, Claessens YE. Presepsin (sCD14-ST), an innate immune response marker in sepsis. Clin Chim Acta. 2015 Oct 23;450:97–103. doi:10.1016/j.cca.2015.06.026 PubMed PMID: 26164388.

135.	Ingham AC, Kielsen K, Cilieborg MS, Lund O, Holmes S, Aarestrup FM, et al. Specific gut microbiome members are associated with distinct immune markers in pediatric allogeneic hematopoietic stem cell transplantation. Microbiome. 2019 Dec;7(1). doi:10.1186/s40168-019-0745-z

136.	Harris AC, Ferrara JLM, Braun TM, Holler E, Teshima T, Levine JE, et al. Plasma biomarkers of lower gastrointestinal and liver acute GVHD. Blood. 2012 Mar 22;119(12):2960–3. doi:10.1182/blood-2011-10-387357 PubMed PMID: 22286196; PubMed Central PMCID: PMC3327467.

137.	Turgunov Y, Ogizbayeva A, Assamidanova S, Matyushko D, Mugazov M, Amanova D, et al. The Role of I-FABP, REG3α, sCD14-ST, and LBP as Indicators of GI Tract Injury in MODS Patients. Diagnostics (Basel). 2025 Feb 20;15(5):515. doi:10.3390/diagnostics15050515 PubMed PMID: 40075763; PubMed Central PMCID: PMC11898716.

138.	Riccardi M, Myhre PL, Zelniker TA, Metra M, Januzzi JL, Inciardi RM. Soluble ST2 in Heart Failure: A Clinical Role beyond B-Type Natriuretic Peptide. J Cardiovasc Dev Dis. 2023 Nov 17;10(11):468. doi:10.3390/jcdd10110468 PubMed PMID: 37998526; PubMed Central PMCID: PMC10672197.

139.	Vander Lugt MT, Braun TM, Hanash S, Ritz J, Ho VT, Antin JH, et al. ST2 as a marker for risk of therapy-resistant graft-versus-host disease and death. N Engl J Med. 2013 Aug 8;369(6):529–39. doi:10.1056/NEJMoa1213299 PubMed PMID: 23924003; PubMed Central PMCID: PMC3943357.

140.	Goldberg RF, Austen WG, Zhang X, Munene G, Mostafa G, Biswas S, et al. Intestinal alkaline phosphatase is a gut mucosal defense factor maintained by enteral nutrition. Proc Natl Acad Sci U S A. 2008 Mar 4;105(9):3551–6. doi:10.1073/pnas.0712140105 PubMed PMID: 18292227; PubMed Central PMCID: PMC2265168.

141.	Liu XC, Li LQ, Ling KR, Guo L, Hu XY, Li C. Fecal HBDay -2 and Claudin-3 may be potential biomarkers to predict the deterioration of necrotizing enterocolitis: A prospective study. Front Pediatr. 2022;10:1062798. doi:10.3389/fped.2022.1062798 PubMed PMID: 36582510; PubMed Central PMCID: PMC9794018.

142.	Rozmus J, Schultz KR. Biomarkers in chronic graft-versus-host disease. Expert Rev Hematol. 2011 Jun;4(3):329–42. doi:10.1586/ehm.11.27 PubMed PMID: 21668397; PubMed Central PMCID: PMC3132532.
